# Ecological drift and host filtering jointly structure foliar endophytes during ecosystem development

**DOI:** 10.1186/s40793-026-00906-7

**Published:** 2026-05-08

**Authors:** Caio César Pires de Paula, Petr Macek, Milan Varsadiya, Jakub Borovec, Mehmet A. Balkan, Brett S. Younginger, Daniel J. Ballhorn, Tomáš Picek, Tomáš Hájek, Jan Frouz, Jiří Bárta, Dagmara Sirová

**Affiliations:** 1 Biology Centre of the Czech Academy of Sciences, Na Sádkách 7, 37005 České Budějovice, Czechia; 2 Faculty of Science, University of South Bohemia, Branišovská 1760, 37005 České Budějovice, Czechia; 3 Department of Biology, Portland State University, P.O. Box 751, Portland, OR 97207-0751 USA; 4 Institute of environmental studies, Faculty of Science, and Charles University Environment centre, Charles University, Benátská 2, 12800 Prague, Czechia

**Keywords:** Community structure, Bacteria, Denitrification, Ecological function, Fungi, Leaf tissue stoichiometry, Primary succession

## Abstract

**Background:**

Foliar endophytes contribute to plant nutrient acquisition, stress tolerance, and pathogen resistance, yet their responses to ecosystem-level processes remain poorly understood. Using a space-for-time substitution design, we investigated bacterial and fungal community dynamics in the foliar endosphere of four phylogenetically distinct plant hosts across a well-characterized successional chronosequence.

**Results:**

Amplicon sequencing revealed that the ecosystem development stage (site age) significantly influenced endophyte community composition, particularly among fungi, but explained only a small proportion of the total variation. Host plant identity and associated leaf stoichiometry were stronger predictors of community structure, with sampling time within the growing season also contributing significantly. Together, these deterministic factors explained 10% and 11% of bacterial and fungal compositional variation, respectively, and 27% of predicted bacterial functional potential. Null model analyses indicated that remaining variation was mostly consistent with stochastic assembly processes, particularly ecological drift. Endophytic communities were characterized by a few persistent dominant taxa and many rare, transient members with overlapping functional potential, including N_2_ fixation, methylotrophy, and denitrification.

**Conclusions:**

Our findings demonstrate that host identity outweighs ecosystem age in structuring foliar endophyte communities and that stochastic processes play a central role in community assembly. The coexistence of stable dominant taxa and a dynamic rare biosphere may enhance plant responsiveness to environmental changes, while the functional potential of endophytes may remain largely consistent across seasons and successional stages.

**Supplementary Information:**

The online version contains supplementary material available at 10.1186/s40793-026-00906-7.

## Background

Endophytic bacteria and fungi inhabit the leaves of all currently described plant phyla across every terrestrial biome [1]. The species-rich and phylogenetically diverse assemblages have been proposed to play key roles in enhancing plant growth, nutrient use efficiency, abiotic stress tolerance, and disease resistance [2–5]. However, comprehensive studies assessing endophytic microbiomes of several taxonomically diverse plant species from a given community are rare [6–8], as are studies focusing on non-model or non-crop plants [9, 10]. Controlled, optimized conditions for the growth of host plants have often been favored over variable, field-realistic conditions in functional studies [4]. Moreover, bacteria and fungi are often studied separately, although they grow together within the endosphere and are likely to interact in important ways [11]. There is currently little information on how ecosystem changes at different spatial and temporal scales affect endophytes or their integrated ecological function. Despite the unforeseen diversity and functional importance of plant-associated endophytes and the rapidly increasing number of studies in this field, we still have limited predictive understanding of the biotic and abiotic factors that govern the microbial communities colonizing plant hosts across ecological development stages, including those created during ecological succession [8].

It is clear that many plant host traits important for success in the early dynamic stages of ecosystem development differ significantly from those that prevail in later successional stages [12]. Published results show that both photosynthetic and transpiration rates generally decrease with successional age, and these differences are reflected in stomatal and mesophyll conductance, which tend to increase with progressing succession [13]. Compared to the shade-acclimated leaves, sun-adapted leaves have been shown to have higher leaf/palisade parenchyma thickness, higher stomatal density, and higher area-based nitrogen (N) content [13]. Previous chronosequence studies have shown that foliar nutrient concentrations often track changes in soil nutrient availability during ecosystem development [14, 15]. All of these changes in leaf architecture and function are likely to shape the physicochemical properties in the foliar endosphere [16]. However, we do not know how strongly the combined effects of the successional stage influence the composition and potential function of foliar endophytes.

Here, we present the structural and functional screening of the foliar endophyte microbiome, based on amplicon sequencing data, in four functionally and taxonomically distinct plant species growing at distinct stages of vegetation succession. We tested the hypothesis that changes in host plant ecophysiology across different stages of ecosystem development will be reflected in the composition, diversity, assembly processes, potential interactions, and ecological functions of foliar endophytic bacterial and fungal communities.

Observing true temporal succession is challenging, and indirect measures, such as chronosequences and associated space-for-time substitutions, are often needed to study successional stages over longer time-scales [17]. Our study area (~ 15 km^2^) is located within the unreclaimed areas of large colliery spoil heaps formed by open-cast coal mining and comprises four vegetated experimental sites in four distinct successional stages on a continuous soil chronosequence [18]. This allowed us to minimize some of the main problems stemming from the use of the space-for-time substitution approach: the four sites differ in age but have nearly identical parent material, topography, climate, and regional species pools [19, 20]. The specific phases of ecosystem development have been well documented at these long-term study sites, revealing strong and predictable shifts in plant community composition, soil microbial community composition, overall ecosystem biodiversity, and ecosystem processes such as primary productivity, biomass accumulation, soil nutrient cycling, and decomposition [21]. Various ecological processes during plant succession have been suggested to be key drivers of leaf elemental ratios [22]. Carbon (C), nitrogen (N), and phosphorus (P) are among the quantitatively most important essential elements and, together with a range of micronutrients, are limiting microbial growth [23]. Hence, we used detailed tissue stoichiometry to characterize host plant leaves as an environment for endophyte colonization.

## Materials and methods

### Study site

The study was conducted at four unreclaimed post-lignite mining locations. The general study area (50° 14′ 21″ N, 12° 40′ 45″ E) is located near the city of Sokolov, Czech Republic, 500–600 m a.s.l., with an average annual precipitation of 650 mm, and a mean annual temperature of 6.8 °C. The parent material at all four sites was homogenized overburden composed of lacustrine clays and claystones (illite, kaolinite, and montmorillonite) and enriched in organic matter (2–18% C), mainly as kerogen. Because of the relatively high abundance of fossil organic matter and mild climatic conditions [24, 25], the heaps offer an opportunity to study ecosystem development-associated changes in a non-extreme environment. The sites are overgrown with vegetation in various stages of spontaneous succession (see below), and the site age (time since overburden heaping) was determined from historical data provided by the coal mining company. Because each successional stage corresponds to a spatially distinct site, ecosystem developmental stage and site identity are inherently confounded in this chronosequence design.

We selected the following four plant species with distinct phylogeny and ecophysiology for leaf endophyte sampling: a perennial grass *Calamagrostis epigejos* (L.), Roth, a deciduous tree/shrub *Salix caprea* L., a coniferous tree *Picea abies* (L.), Karst., and a perennial herb *Tussilago farfara* L. All four species were present at all four selected sites (10, 20, 30, and 54 years after overburden deposition), designated here as I, II, III, and IV, respectively (Fig. S1). The four sites are arranged in an approximate linear sequence, spanning ~ 7 km from site I to site IV (Fig. S1). The vegetation changed from herb-dominated at the earliest successional stage through shrub-dominated to forest-dominated at the most advanced successional stage [19, 20]. *Calamagrostis epigejos* dominated at the two youngest sites (I, II), interspersed with *S. caprea* and *P. abies* individuals (reaching only shrub height). The 30-year-old site (III) harbored a transitional forest with *S. caprea* as the dominant tree species and sparse populations of the other three studied species. The site with the most advanced stage of vegetation succession contained a developed forest in which the dominant trees were *Betula pendula* Roth and *Populus tremula* L., while *C. epigejos* dominated the understory; *S. caprea* and *P. abies* populations were sparse [19, 20].

### Soil analysis

In May 2017, we collected soil samples (after litter removal) from 0 to 5 cm depth. Four samples from distinct spots at least 10 m apart were collected at each location. The soil was immediately transported to the laboratory, homogenized, and passed through a 2 mm mesh, yielding 16 individual samples, each weighing approximately 200 g. Part of each sample was air-dried for chemical analyses. The rest was processed immediately for microbial C content determination (using the fumigation extraction method [26]). Soil pH was determined in water suspension (1:5 ratio) using a glass electrode. Soil C, N, and P total content was measured as described below for leaf tissue samples. The microbial C and N content in extracts was measured, using the standard protocol, on the OC-LCPH/CPN analyzer, Shimadzu Corp., Kyoto, Japan.

### Leaf sampling

In the first week of May 2017 (spring, the start of the growing season), six individuals from each species and each successional stage growing at least 15 m apart were selected and marked for the next sampling point in the season [18]. From each plant individual, two (*T. farfara* and *C. epigejos*) to six (*S. caprea*) fully developed leaves of the same age without visible herbivore damage or disease lesions, were collected. For *P. abies,* six needles from four branches from the 2016 growth were collected. For the two tree species, samples were collected from different sides of the plant, at the same height from the ground, and at the same leaf position (to minimize confounding effects). Leaf petioles were retained. To ensure the true endophyte community is analyzed, collected leaves were surface-sterilized in the field [27] and immediately placed into liquid nitrogen. The efficacy of surface sterilization was tested periodically by inoculating sterilized leaf imprints onto agar plates, and the results were negligible. Following transport to the laboratory, leaf samples were finely ground under liquid nitrogen using a sterile mortar and pestle, then stored at − 80 °C until further analysis. All leaves collected from a single plant individual were pooled to form a composite sample. This sampling scheme was repeated in the first week of August (summer, peak of the growing season) and the second week of October 2017 (autumn, beginning of senescence) with identical plant individuals.

### Leaf tissue chemistry analyses

To be used as a proxy for characterizing the conditions in the environment colonized by foliar endophytes, we analyzed leaf tissue elemental content in each sample. Total C and N contents (primary nutrients) in freeze-dried and homogenized leaf tissue were analyzed on the CHNS analyzer Vario MICRO Cube (Elementar, Germany). Total P content and the contents of other essential macronutrients with a possible effect on microbial growth, including potassium (K), magnesium (Mg), calcium (Ca), sulfur (S), and trace metals, were determined using the ICP-QQQ (Agilent, Japan), following wet digestion by HClO_4_ (at 170 °C for 2 h). Contents were expressed as µg^−1^ dry tissue.

### DNA extraction and sequencing

Approximately 100 mg of homogenized leaf tissue was used for DNA extraction with the DNeasy Plant Mini Kit (Qiagen, Hilden, Germany) following the manufacturer’s protocol [18]. In total, 272 leaf samples were successfully extracted and sequenced. The V3–V4 region of the bacterial small-subunit (*16S*) *rRNA* gene and the internal transcribed spacer region (ITS) of the ribosomal operon were PCR amplified using universal primers for bacteria (335F: 5′-CADACTCCTACGGGAGGC-3′/769R: 5′-ATCCTGTTTGMTMCCCVRRC-′) [28, 29] and fungi (ITS1: 5′-CTTGGTCATTTAGAGGAAGTAA/ITS2: 5′-GCTGCGTTCTTCATCGATGC-3′) [30]. The extracted DNA was amplified using the PPP Master Mix Polymerase 2× concentrated (TOP-BIO, Czech Republic) in a final volume of 20 μL per sample (16S: 3 min at 95 °C/30 cycles: 30 s at 95 °C, 15 s at 60 °C, 40 s at 72 °C/10 min at 72 °C; ITS: 3 min at 94 °C/30 cycles: 15 s at 94 °C, 15 s at 60 °C, 45 s at 72 °C/10 min at 72 °C). Amplicons were sent to the DNA Services Facility at the University of Illinois (Chicago, USA) for sequencing using the Illumina MiniSeq MO (2 × 150) and the Illumina MiSeq V2 (2 × 250) platforms (Illumina Inc., United States) for 16S and ITS amplicons, respectively. Negative extraction and PCR controls were included at all steps and sequenced alongside leaf tissue samples. The raw sequences have been deposited in the NCBI Sequence Read Archive under the BioProject accession numbers PRJNA894138 (16S) and PRJNA894357 (ITS).

### Sequence analysis

The raw reads were processed using DADA2 [31] and the R Software (v4.0.2; R Core Team, 2020). First, *cutadapt* v1.12 [32, 33] was used to remove the primers from both the forward and reverse sequences. Then, the sequence reads were filtered with an expected error threshold of 2 and trimmed to 250 and 200 bases (16S rRNA) or 350 and 300 bases (ITS) for the forward and reverse reads, respectively. Filtered reads were then de-replicated and de-noised using DADA2 default parameters. De-replication combines identical reads into unique sequences and constructs quality profiles for each combined sequence. After merging the forward and reverse sequences, the taxonomic classification of amplicon sequence variants (ASVs) was obtained using the Silva 138 [34] and UNITE 7.2 [35] databases for 16S rRNA and ITS sequences, respectively. Prokaryotic sequences that were identified as originating from organelles (chloroplasts, mitochondria), as well as eukaryotic sequences identified as originating from animals (Metazoa), plants (including algae), or of unknown eukaryotic origin, were removed before data analysis. A total of 57,014,224 high-quality bacterial and 5,476,826 fungal sequences were obtained (Table S1). The average per sample was 21,038 ± 7617 16S rRNA sequences and 20,135 ± 11,134 ITS sequences. Bacterial and fungal sequences were clustered into 7695 and 6865 ASVs, respectively, representing 29 bacterial and 6 fungal phyla. Among the fungal ASVs, 333 (4.85%) and 2763 (40.26%) were unidentifiable at the phylum and genus level, respectively. In contrast, all bacterial ASVs were assigned at the phylum level, and 18.99% (1 462 ASVs) were unidentifiable at the genus level.

### Turnover rates of endophyte taxa

Changes in the relative abundance of bacterial and fungal genera between two successive sampling points during the growing season were evaluated according to published methodology [36]. We then calculated taxa turnover according to the following formula: ([number of genera gained] + [number of genera lost])/(total number of genera observed at both sampling points combined) [37]. This calculation is based on the original formulation [38], which has subsequently been modified [39] to allow comparisons of proportional turnover between locations differing in starting species richness.

### Functional assignment of bacterial genera

We mined the amplicon sequencing data for a more ecologically meaningful insight and a more biogeochemistry-focused overview of the microbiome functional potential by classifying the presence/absence of a selected suit of functional genes within the genomes of bacterial genera identified in this study. We compared our data with the curated information in the Functional Gene Pipeline and Repository (FunGene) [40]. Functional inference was limited to bacterial taxa because available tools (e.g., FunGene) provide gene-based functional annotations for bacteria, whereas current fungal databases (e.g., FUNGuild, FungalTraits) [41] rely largely on ecological guild classifications rather than verified gene presence, resulting in lower functional resolution. The lists of bacterial genera for each individual gene were downloaded directly from the FunGene database. A condensed list of unique genera was created and used to annotate functions in an ASV table. The relative proportion of genera with known presence of a specific functional gene in the genome within a given sample was used for among-sample comparisons. The following genes linked to major N biogeochemical pathways [40] were selected for analysis: *nifH* and *nifD* (N_2_-fixation); *napA* and *narG* (reduction of nitrate to nitrite); *nirA* and *nirK* (reduction of nitrite to nitrous oxide); *nor*B (oxidation of nitrous oxide to nitric oxide); *nrfA* (direct reduction of nitrite to ammonia); *ureA* (conversion of urea to ammonia); *amoA* (ammonia oxidation). These were complemented by genes used as markers for complex organic matter degradation: *chB* (chitin degradation), ligE (lignin degradation), and *lacc* and *ppo* (degradation of phenolic substances). Also included were genes involved in sulfur metabolism (*dsrA* and *dsrB*—sulfite and sulfate reduction, respectively; *soxB*—sulfur oxidation), as well as methylotrophy and methanotrophy (*mmoX* and *pmoA*, respectively). Because functional annotation was based on genus-level presence of genes reported in reference genomes, these results represent potential functional capacity rather than confirmed gene presence in the studied communities.

### Network construction

To better understand microbial interactions within the foliar endosphere, we constructed genus-level cross-domain co-occurrence networks. To increase their robustness and reduce spurious correlations caused by low-abundance taxa [42], we used only genera that were present in at least 5% of samples and had more than 10 reads. The Sparse Correlations for Compositional data (SparCC) algorithm was used to infer correlations using C++ implemented FastSpar [43]. Absolute correlation coefficients ≥ 0.3 were retained. The fastspar_bootstrap() function with 100 replicates was used to generate pseudo-*p* values, and only edges whose *p* value was ≤ 0.05 were retained. Network images were generated in R using the *Igraph* package. In the network, nodes represent genera, whereas edges represent correlations between nodes. We used the undirected network approach (in which edges are undirected) and the Fruchterman–Reingold layout. The topology properties of the co-occurrence networks, positive edge, negative edge, total node, average path length (APL), clustering coefficient (CC), and number of modules were calculated using the *igraph* R package [44].

Two parameters described different topological roles of individual genera. The first was the within-module connectivity (*Zi*), which describes how well a node is connected with other nodes within its module. In contrast, the second parameter—connectivity among modules (*Pi*)—suggests how well a node connects to different modules. The threshold values for *Zi* and *Pi* in node categorization were 2.5 and 0.62, respectively, according to previous studies [45]. In general, the topological role of each node was subdivided into four categories according to ecological strategy [46]: (1) peripheral nodes (specialists, low *Zi* (< 2.5) and *Pi* value (< 0.62), i.e., few edges and only two nodes within their modules); (2) connectors (generalists, low *Zi* (< 2.5) but a high *Pi* value (> 0.62), i.e. more connections with several modules); (3) module hubs (generalists, high *Zi* (> 2.5) but a low *Pi* value (< 0.62), i.e. nodes have more connections with other nodes within their module only); 4) network hubs (supergeneralists, high *Zi* (> 2.5) and *Pi* (> 0.62) values, i.e. connector and module hubs). Generalists and supergeneralists are considered key taxa within co-occurrence networks, maintaining their stability and performing key ecological functions [47].

### Community assembly processes

To determine the dominant processes shaping the endosphere microbiome, we used a published approach [48], where assembling processes are classified into the following categories: (1) homogeneous selection (abiotic or biotic pressures select for the same types of characteristics across communities); (2) variable selection (abiotic or biotic pressures select for different types of characteristics across communities); (3) homogenizing dispersal (individuals can move between communities easily); (4) dispersal limitation (individuals cannot move between communities easily), and (5) drift (population fluctuations are essentially due to weak selection, weak dispersal and/or random chance events) [48, 49]. Briefly, for a given pair of samples, we calculated phylogenetic dissimilarity between them using *β*-mean-nearest-taxon-distance (*βMNTD*) and its deviation from the mean of the null distribution and evaluated significance using the β-Nearest Taxon Index (*βNTI*; the difference between observed *βMNTD* and the mean of the null distribution, in units of standard deviation). If the observed *βMNTD* value is significantly greater (*βNTI* > 2) or smaller (*βNTI* < − 2) than the null expectation, the community is considered to be assembled by variable or homogeneous selection, respectively. If there is no significant deviation from the null expectation, the observed differences in phylogenetic community composition should result from dispersal limitation, homogenizing dispersal, or random drift.

In the second step, the abundance-based (Raup-Crick) beta-diversity was calculated using pairwise Bray–Curtis dissimilarity (*βRC*) [50, 51] to estimate the relative importance of these processes. For this analysis, we used the *raup_crick* function [50, 51]. Based on the calculated *β*_*RC*_, we can assume that communities that were not selected in the first step, thus not assembled by selection, were structured by (1) dispersal limitation if *β*_*RC*_ > + 0.95, (2) mass effect if *β*_*RC*_ < − 0.95, or (3) stochastic processes consistent with ecological drift if *β*_*RC*_ falls in between − 0.95 and + 0.95. The relative importance of a process was measured as the percentage of comparisons dominated by each process.

### Statistical analyses

Statistical analyses and graphs were generated using R Software (v4.0.2; R Core Team, 2020), MicrobiomeAnalyst tools [52], CANOCO 5 [53], and Past 4.14 [54]. Stoichiometry data were tested for normality and homogeneity of variances using the Shapiro–Wilk test; in cases of non-normality and/or heteroscedasticity, the data were log_10_-transformed. Prior to statistical analysis, microbial data were preprocessed to ensure data comparability: taxa were retained if present (i.e., non-zero counts) in at least 10% of samples. After prevalence filtering (≥ 10% of samples), data were CLR-transformed to account for compositional structure [55]. At first, we tested the effects of ecosystem developmental stage, species, and time on foliar stoichiometry using variation partitioning [55] combined with RDA [54] to assess the unique contributions of each variable and the shared parts of all possible variable combinations. To explain variation in endophyte community composition, we again applied variation partitioning [53, 56], using plant species identity, developmental stage, and season as explanatory variables. The conditional effects (variability explained by a single variable when the other two were used as covariates) and shared variability explained by the compositional and stoichiometric data were tested. Data were log-transformed, and ASVs with occurrences ≤ 3 were excluded from the analyses. The effect of stoichiometric data on the variation of endophyte communities was also tested by the same analysis using the following factors: primary nutrients (C, N, P, C/N, C/P), essential macronutrients (Mn, K, Ca, S, Mg, B), and trace metals (Fe, Zn, Al, Ni, Mo, Co, Hg, Pb, As, Cr). Finally, the effect of the above explanatory variables on the functional composition of bacterial endophyte communities was tested by variation partitioning followed by forward selection (499 Monte Carlo permutations) of the most important (significant) elements. Bacterial and fungal richness (Chao1) and the α-diversity (Shannon–Weiner Index) were estimated using the *phyloseq* package (v1.32) [56]. Differences in alpha-diversity indices (Chao1 and Shannon–Weiner Index) and environmental stoichiometry (C:N, C:P and N:P) were tested across successional stages, seasons, and plant species using three-way factorial analysis of variance (ANOVA). This approach allowed for the evaluation of both main effects and their interaction terms. When significant effects were detected, post-hoc comparisons were performed using Estimated Marginal Means (EMMeans) to identify specific differences between locations for individual species, and between species within a given location using *emmeans* package. *P* values were adjusted for multiple comparisons using the Tukey method. To evaluate temporal shifts for specific plant species, paired t-tests were utilized to compare turnover between time points. To assess how these rates varied spatially and across hosts, Kruskal–Wallis tests were performed, followed by Dunn’s post hoc test with Bonferroni correction to identify significant differences in turnover for individual species across the four locations, as well as differences among the four plant species within the same location. The correlation between most abundant taxa and stoichiometric data was obtained using *microbiomeSeq* package [57], and the associated *p* values were adjusted for multiple testing using the Benjamin and Hochberg method. Principal coordinate analysis (PCoA) with Bray–Curtis dissimilarities was used to assess the similarity or heterogeneity of microbial community composition (β-diversity) across all four sampled sites at the three sampling points during the growing seasons. The ordination analysis was coupled with permutational multivariate analysis of variance (PERMANOVA) to statistically assess differences in community composition among plant species, using *F*-statistics, *R*^*2*^ values (explained variance), and associated *p* values. The linear discriminant analysis (LDA) effect size (LEfSe) method was used to identify the genera most likely to explain differences between sample groups by coupling standard statistical significance tests with additional tests assessing biological consistency and effect relevance [58]. The threshold on the logarithmic LDA score for discriminative features was set at 2.0 and *α* = 0.05, and features with at least 2.0 log-fold changes were considered significant. The bacterial and fungal genera shared between plant species and across successional stages and seasons were generated using Venny 2.1 (https://bioinfogp.cnb.csic.es/tools/venny/index.html) and visualized with the ggvenn package (v. 0.1.10) [59].

## Results

### Location characteristics and leaf tissue chemistry

Soil nutrient concentrations peaked at the transitional forest (III), particularly total N, organic C, and microbial biomass N and C, whereas pH, conductivity, and total P did not differ significantly among sites (Table S2).

**Fig. 1:**
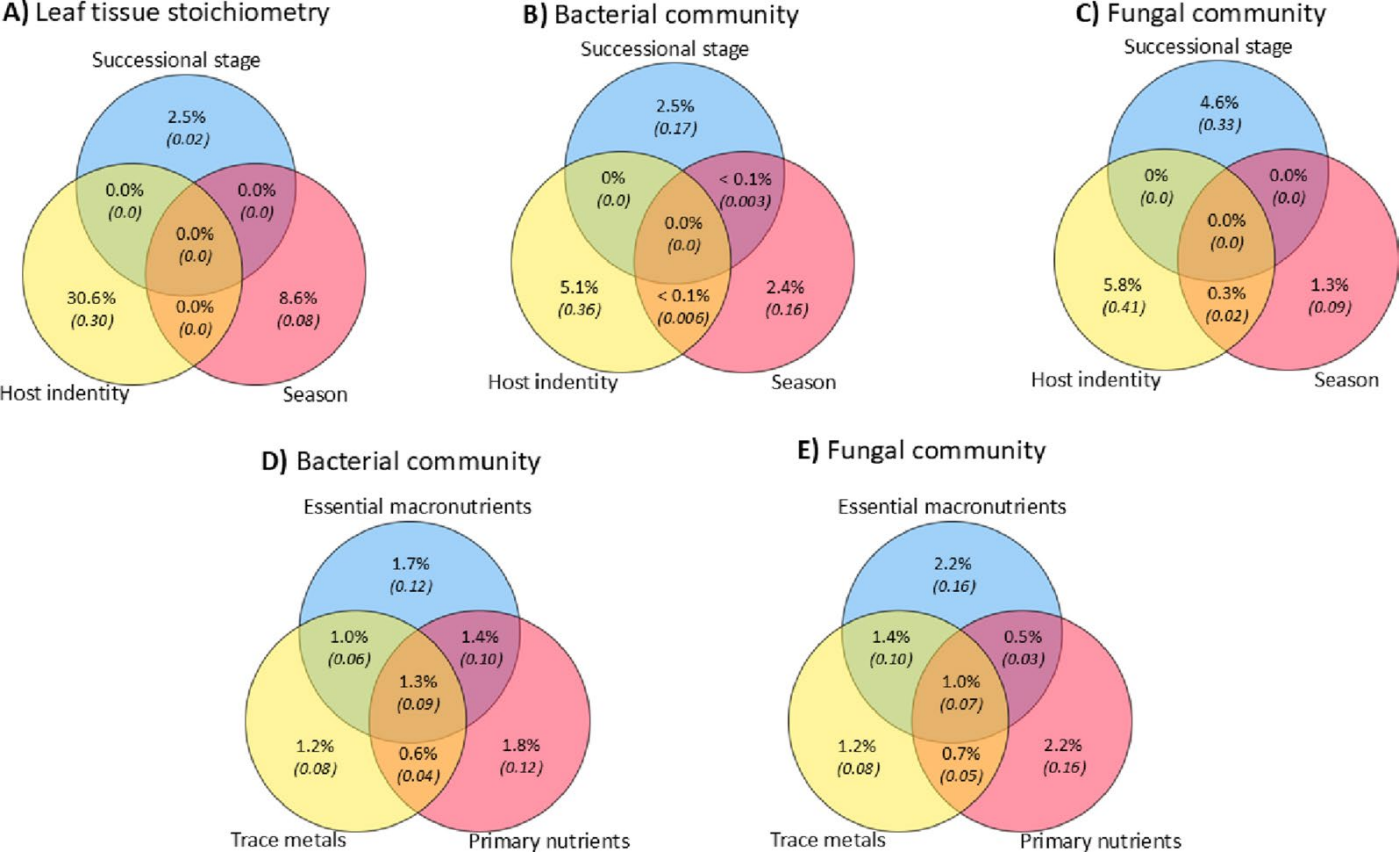
Venn diagrams showing the contribution of host identity, successional stage, and season, as well as their interactions, to the variation in **A** leaf tissue stoichiometry, **B** bacterial, and **C** fungal community structure. The same analysis also depicts the importance of essential macronutrients, trace metals, and primary nutrients in explaining the variation of **D** bacterial and **E** fungal community structure. Values on each diagram represent the fraction’s contribution to the total variation in the dataset, while values in parentheses indicate the adjusted R^2^, i.e., the proportion of variation uniquely explained by each fraction after accounting for shared effects and model complexity

Variation partitioning analysis showed that the leaf tissue chemistry was significantly affected by the successional stage, season, and species (2.5%, 8.6%, and 30.6% of total variability explained, respectively; Fig. 1A, Fig. S2, Table S3). In general (Table S4 for averages and statistics, Table S5 for detailed data), the C:N and C:P ratios were highest in *P. abies*, with a distinct N content rise in autumn at all four successional stages, which can be attributed to antifreeze protein accumulation in the intercellular spaces of the needles during cold acclimation [60]. In *P. abies*, the summer and autumn C:N ratios were significantly higher at the two earliest successional stages than the later successional sites (*p*_CN−summer_ < 0.0001; *p*_CN−autumn_ < 0.007). In the two pioneer species, *S. caprea* and *T. farfara*, the C:N and C:P ratios increased uniformly from spring to autumn, and differences among sites at different stages of ecosystem development were largely insignificant. The C:N ratios in the grass *C. epigejos* were highest in summer. Location II, where *C. epigejos* had the highest coverage, also had significantly higher C:N ratios than the other three sites.

### Effect of plant host species on foliar endophyte community composition

**Fig. 2:**
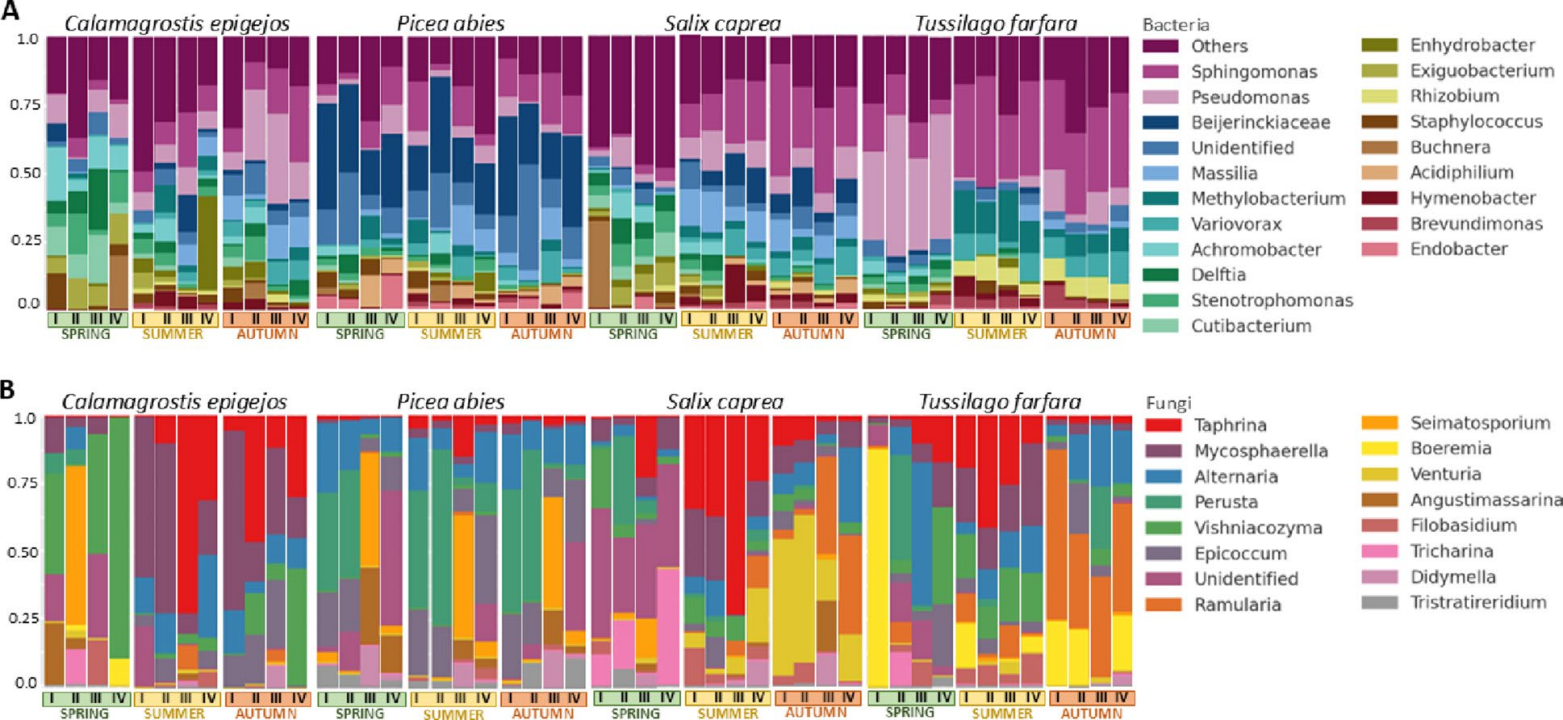
Bacterial (**A**) and fungal (**B**) composition profiles. The most abundant taxa detected in the leaf endosphere of four studied plant species (*Calamagrostis epigejos*, *Picea abies*, *Salix caprea*, and *Tussilago farfara*) growing at different stages of ecological succession (locations I, II, III, and IV), during a single growing season (spring, summer, and autumn) are shown

The plant host species significantly affected the structuring of foliar endosphere bacteria and fungi (5.1% and 5.8% of total variability explained, respectively; Fig. 1B, C, Table S3). However, when considering the 20 most abundant bacterial taxa, the foliar endosphere communities of all four host plant species were highly similar (Fig. 2A; Table S1).

The bacterial dataset was dominated primarily by Proteobacteria, with genera such as *Achromobacter*, *Methylobacterium*, *Sphingomonas*, *Massilia*, *Pseudomonas*, and *Variovorax* occurring at high relative proportions across hosts (Fig. 2A; Table S1). Bacterial richness was significantly lower in *C. epigejos* than in the other three species (Table S6), whereas Shannon diversity did not differ significantly in the two pioneers, *S. caprea* and *T. farfara.* The two trees, *P. abies* and *S. caprea* showed the highest diversity in the later successional stages (Table S7).

In fungal endophyte communities, several Ascomycete genera—including *Alternaria*, *Epicoccum*, *Mycosphaerella*, and *Taphrina*—were abundant in most samples (Fig. 2B; Table S1). As in bacteria, host identity significantly affected fungal community diversity and richness (Tables S8–S9). Overall, fungal diversity tended to be higher in the two tree species than in the herbaceous hosts (Table S9). Notably, *P. abies* needles in spring exhibited significantly higher fungal richness and diversity than those of the other plant hosts, likely reflecting the presence of overwintering taxa.

The proportion of endophytic genera shared by all four host species was generally low, particularly in spring (< 10%), and averaged approximately 12% for bacteria (Fig. S3) and 13% for fungi (Fig. S4) in summer and autumn. *Tussilago farfara* harbored the highest proportion of unique bacterial and fungal genera during most sampling periods (up to 36.0%; Figs. S3–S4). At the later successional stages (locations III and IV), a significantly higher proportion of bacterial taxa was shared among all host species compared to the early successional stages (locations I and II; *p* = 0.01, Fig. S3), suggesting increased community convergence with ecosystem development.

### Effect of successional stage on foliar endophyte community composition

The endosphere microbiome composition was significantly influenced by ecosystem developmental stage (Fig. 1B, C, Table S3). The effect was stronger for fungal than bacterial communities (4.6% vs. 2.5% of total variability explained, respectively), although overall effect sizes were modest relative to host identity. Bacterial richness and diversity increased from the earliest successional stage, peaked at the transitional forest stage (site III), and declined slightly at the most advanced stage (Fig. S5; Tables S6 and S7). In contrast, fungal community diversity was generally higher at the later successional stages characterized by a developed canopy layer (sites III and IV; Fig. S5; Table S9). These trends, however, were host-specific.

Mean bacterial diversity values were approximately half of those previously reported for soil communities at the same locations, whereas fungal diversity was comparable to that reported for bulk soil at these sites [21].

### Effect of season on foliar endophyte community composition

Season significantly influenced endosphere community composition, although it explained a relatively small proportion of total variability (2.4% in bacteria and 1.3% in fungi; Fig. 1B and C, Table S3).

**Fig. 3:**
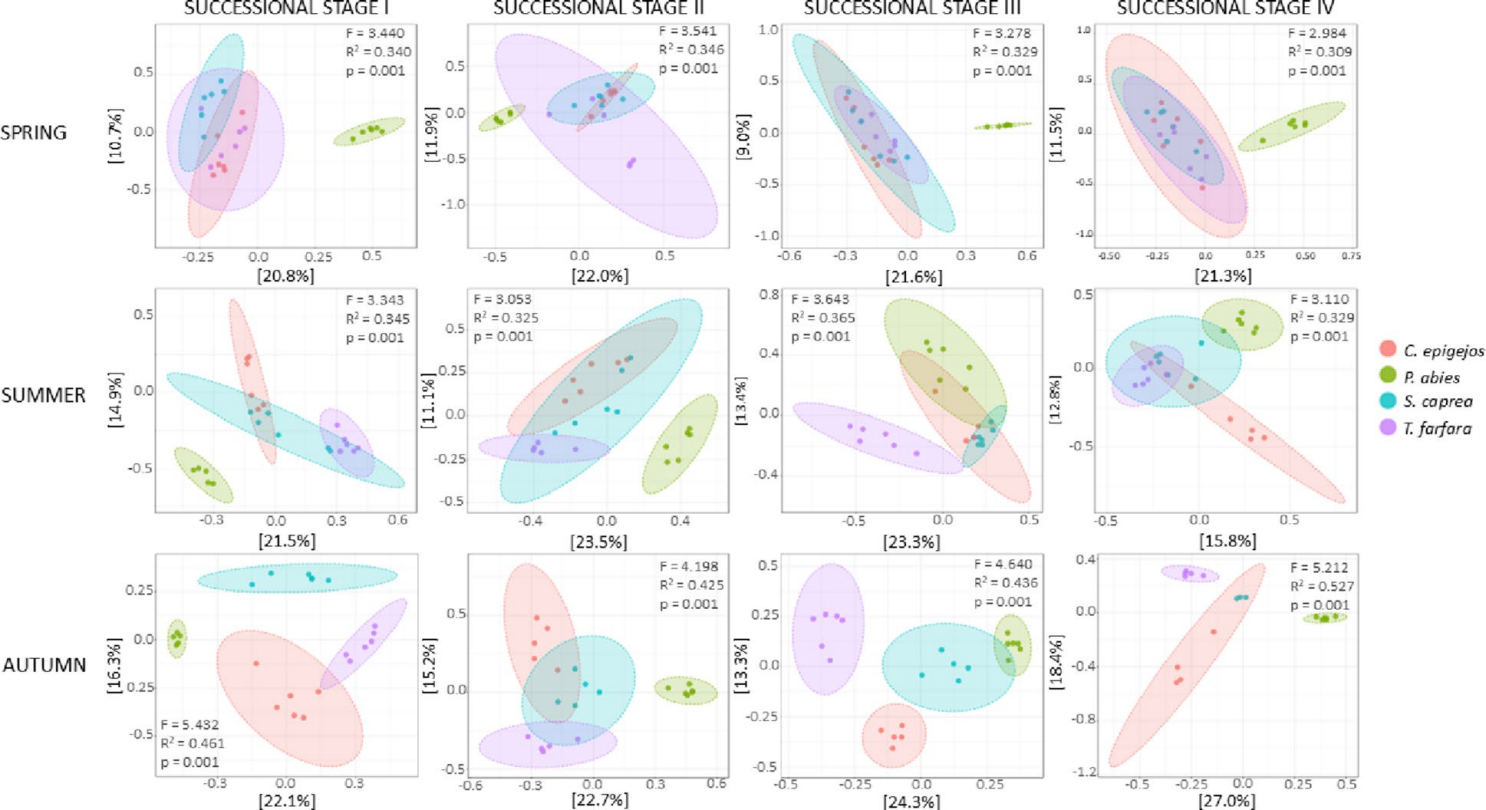
Principal coordinates analysis (PCoA; Bray–Curtis distance) plot visualizing the *ß*-diversity of the foliar endosphere microbiomes (bacteria and fungi together, at the ASV level) of the four studied plant host species (*Calamagrostis epigejos*, *Picea abies*, *Salix caprea*, and *Tussilago farfara*), growing at different stages of ecological succession (I, II, III, and IV) during a single growing season (spring, summer, and autumn). Ellipses indicate a 95% confidence interval. Each dot represents the foliar endosphere microbiome of a single plant individual. *F*-statistics, R^2^ values, and associated *p* values (PERMANOVA) are displayed

The structure of microbial communities showed distinct host-specific patterns that diverged over time (Fig. 3). While the three deciduous hosts (*C. epigejos*, *S. caprea*, and *T. farfara*) exhibited significant similarity in spring, their microbial communities became increasingly distinct as the growing season progressed, reaching maximum differentiation in autumn. In contrast, the evergreen *P. abies* formed a distinct cluster across all successional stages and seasons, indicating a more stable community structure, likely associated with the persistence of overwintering taxa.

### Temporal turnover of endophyte genera

**Fig. 4:**
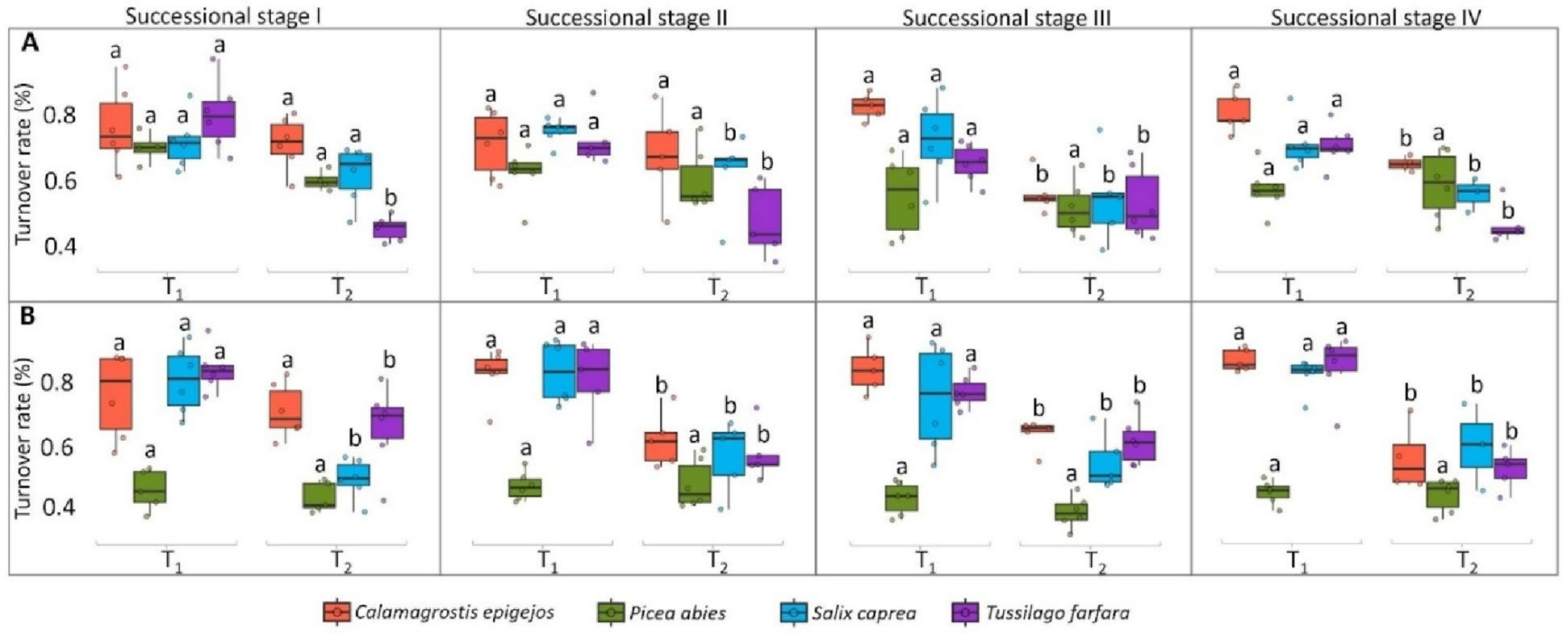
Temporal turnover of **A** bacterial and **B** fungal endophytes in *Calamagrostis epigejos*, *Picea abies*, *Salix caprea*, and *Tussilago farfara*. Shifts in the composition of endophyte taxa were analyzed across all successional stages (I, II, III, and IV) for two periods: spring to summer (T1) and summer to autumn (T2). Different letters indicate a significant difference in turnover rates for the same species at the same location within a successional stage

Turnover rates of bacterial and fungal endophytes were high and variable across hosts and sites (Fig. 4A, B; Table S10). Between spring and summer, bacterial turnover was lowest in *P. abies*, particularly at later successional stages, and higher in the herbaceous hosts. Turnover rates declined significantly from summer to autumn (*p* < 0.001) for bacterial and fungal endophytes across all hosts except *P. abies*. Fungal communities exhibited similar temporal patterns, with slightly higher overall turnover than bacterial communities. From spring to summer, fungal turnover in *P. abies* was approximately half that observed in the other host species.

### Effect of foliar tissue stoichiometry on foliar endophyte community composition

Leaf tissue chemistry significantly influenced both bacterial and fungal endophytes, explaining 9.3% and 9.1% of total variability, respectively (Fig. 1D, E; Table S3). In both groups, total C and N content, C:N ratio, and selected macronutrients and trace elements (notably K and Ca for bacteria, and S for fungi) were among the strongest predictors.

Responses to tissue stoichiometry were highly ASV-specific. Closely related taxa frequently exhibited contrasting associations depending on the host species and season (Fig. S6). For example, different ASVs assigned to the same genus (e.g., *Sphingomonas* or *Ramularia*) showed opposite correlations with leaf C:N ratios across hosts, highlighting fine-scale ecological differentiation within genera.

### Profiles of potential function in bacterial foliar endophytes

Ecosystem developmental stage, season, and host identity together explained 27.4% of variation in predicted functional gene profiles (Table S3). Host species identity accounted for the largest proportion of explained variation (23.1%), followed by the season (4.0%) and the successional stage (0.7%).

**Fig. 5:**
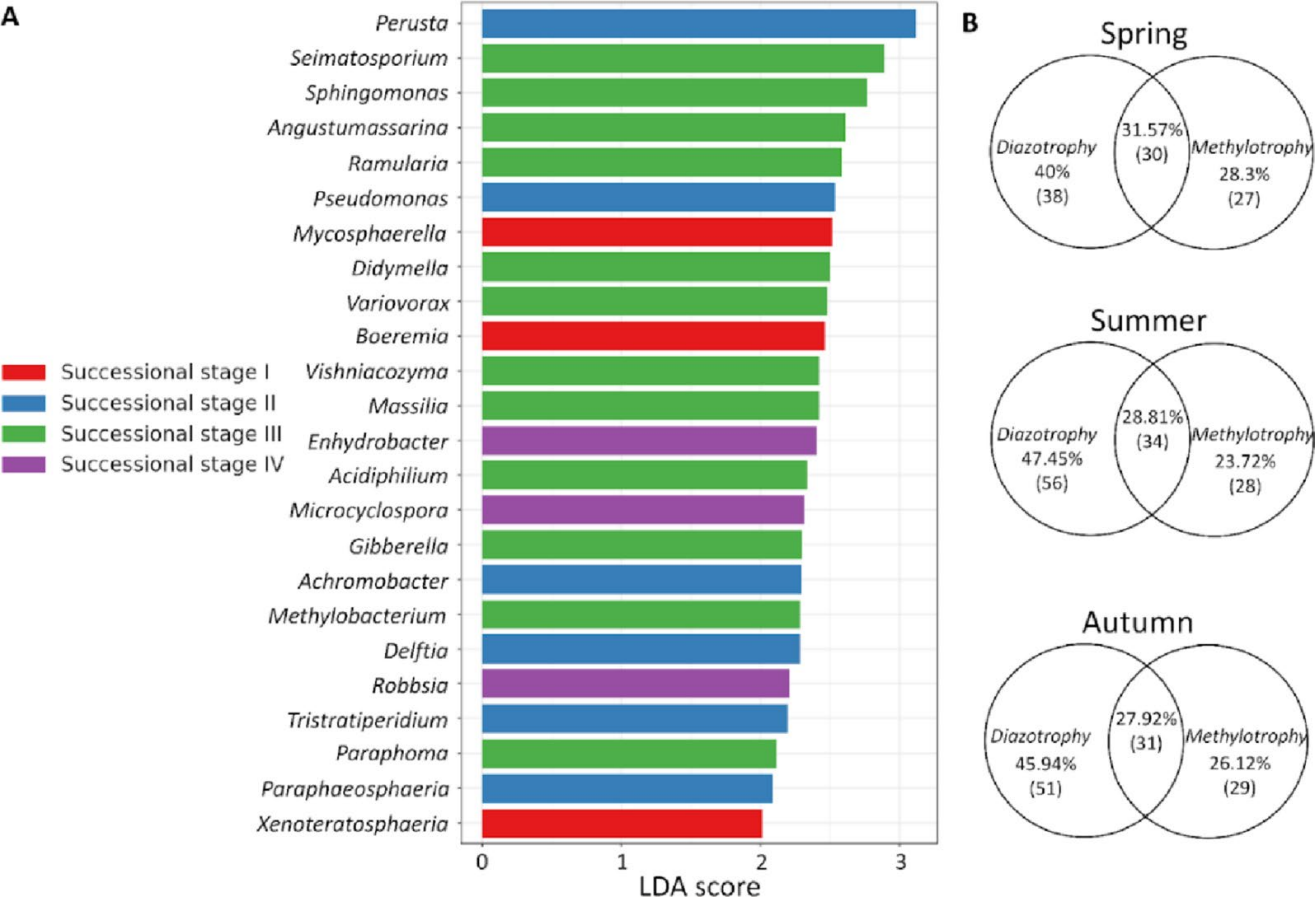
**A** Linear discriminant analysis (LDA) effect size (LEfSe) displaying significant indicator taxa at the genus level, present in the foliar endophere at the four successional stages (I, II, III, and IV). Bar graphs represent the LDA score. **B** Proportion and total cases (in parentheses) of bacterial genera with the potential for both diazotrophy (N₂-fixation) and methylotrophy, present across all studied plant host species during spring, summer, and autumn

Across all hosts and successional stages, endophyte communities contained a high relative proportion of genera known to harbor genes associated with atmospheric N-fixation (Fig. 5). The relative importance of potential diazotrophs increased toward the end of the growing season and averaged approximately 50–60% of the community across hosts (Table S11). Methylotrophic genera were also abundant, with cumulative relative proportions reaching high levels in autumn samples. A substantial fraction of taxa was potentially capable of both diazotrophy and methylotrophy, and this proportion remained relatively constant throughout the year despite the shifts in overall community composition (Fig. 5).

Genera implicated in additional N-cycling pathways—including dissimilatory nitrate reduction, denitrification, and urea metabolism—were consistently detected at high relative proportions, whereas known ammonia oxidizers were rare and nitrite oxidizers were not detected. Taxa with genomes known to encode enzymes involved in the degradation of complex organic substrates were also widespread, particularly in *T. farfara*.

### Potential indicator taxa

LEfSe analysis identified 24 genera that discriminated among successional stages (Fig. 5). Most early-stage indicators were fungal, whereas taxa characteristic of the intermediate stage included both bacterial and fungal genera. Fewer indicator taxa were associated with the most advanced successional stage.

Among the plant hosts and across seasons, 16 bacterial and 21 fungal genera were identified as discriminative taxa (LDA score > 2.0; Fig. S7). *Tussilago farfara* harbored the highest number of bacterial biomarkers, whereas *P. abies* contained the largest number of fungal indicators. The number of season-specific biomarkers increased from spring to autumn.

### Cross-domain co-occurrence network analysis

**Fig. 6:**
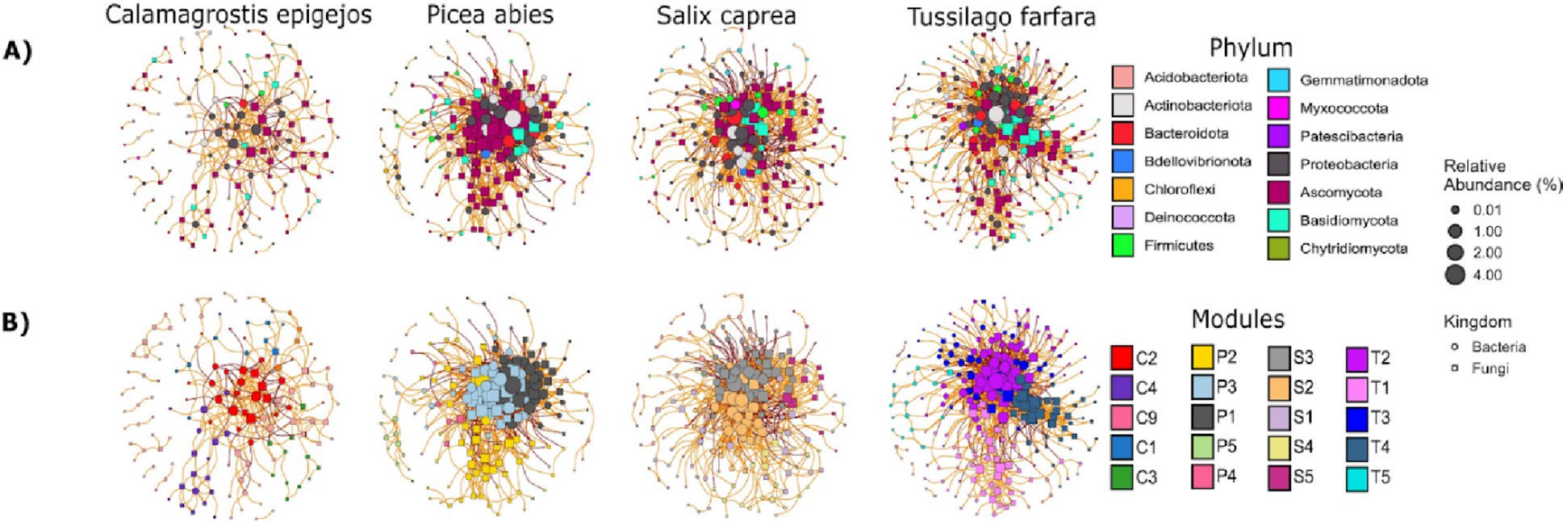
Cross-domain co-occurrence network showing microbial interactions for each host plant species (*Calamagrostis epigejos*, *Picea abies*, *Salix capre*a, and *Tussilago farfara*). The upper panel (**A**) displays the main phyla belonging to the respective modules shown in the bottom panel (**B**). Each symbol represents a genus—circles for bacteria and squares for fungi—and symbol size indicates relative abundance

Cross-domain co-occurrence network analysis revealed pronounced host-specific differences in network structure (Fig. 6; Table S12). Network complexity, reflected by connectivity and clustering coefficient, was highest in *P. abies* and lowest in *C. epigejos*, while the node number and the proportion of connected genera were highest in the two pioneer species, *T. farfara* and *S. caprea*.

Season strongly influenced network architecture. Spring networks showed high connectivity in *P. abies* and *S. caprea*, whereas summer networks were most complex in *S. caprea* and *T. farfara*. Positive correlations generally outnumbered negative ones across all species and successional stages. The ratio of positive to negative interactions was lower in summer, suggesting a seasonal transition from early-season facilitation toward more complex interactions such as competition or niche partitioning. The network structure shifted from summer to autumn, with the representation of fungal taxa increasing across most hosts. In *P. abies*, fungal nodes consistently outnumbered bacterial nodes, possibly reflecting the stable, more strongly developed apoplast communities colonizing evergreen hosts compared with deciduous hosts.

Successional progression and seasonal shifts significantly altered the taxonomic composition of the networks. Early successional stages and spring samples were primarily enriched in Proteobacteria, whereas later successional stages and autumn networks showed an increased proportion of fungal taxa, particularly Ascomycota and Basidiomycota.

Most nodes were classified as peripherals (92.70%), with relatively few connectors (6.38%), module hubs (0.65%), and network hubs (0.26%; Fig. S8). *Tussilago farfara* harbored the highest numbers of connectors and hubs. Genera associated with N_2_-fixation, methylotrophy, and potential plant pathogenicity were common structural components across the networks. Seasonal shifts were also evident, with methylotrophs more prominent in spring and fungal saprotrophs enriched in summer and autumn.

### Main assembly processes in foliar endosphere microbiomes

**Fig. 7:**
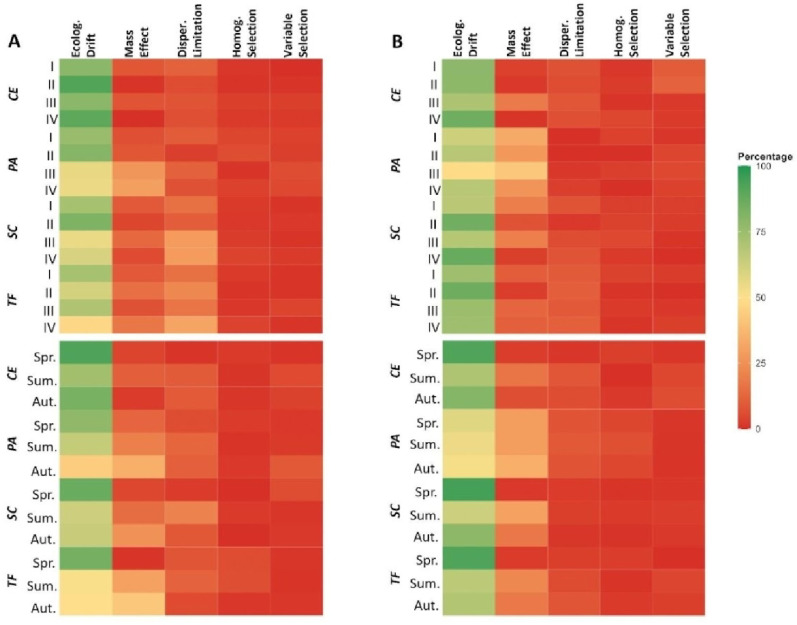
Relative importance of high-level processes structuring the bacterial (**A**) and fungal (**B**) metacommunity in the leaf endosphere of four studied plant species (*Calamagrostis epigejos—‘CE’*, *Picea abies—‘PA’*, *Salix caprea—‘SC’*, and *Tussilago farfara—‘TF’*) growing at different stages of ecological succession (locations I, II, III, and IV), during a single growing season (spring—‘Spr.’, summer—‘Sum’, and autumn—‘Aut’). The values at the scale bar indicate percentage of community assembly associated with each process: homogeneous selection (*βNTI* < − 2), variable selection (*βNTI* > + 2), mass effect (RC_bray_ < − 0.95), dispersal limitation (*RC*_bray_ > + 0.95), and ecological drift (|*RC*_bray_|< 0.95)

Null model analyses indicated that stochastic processes dominated endophyte community assembly across all hosts and successional stages. The *βNTI* values suggested that stochastic dynamics accounted for more than 90% of community turnover in all plant species. When combined with Raup–Crick metrics, ecological drift emerged as the primary assembly process (Fig. 7).

*Picea abies* exhibited a slightly lower contribution of drift and a higher proportion of mass effects relative to the other hosts. Dispersal limitation increased along the successional gradient, particularly in bacterial communities, whereas selection played a somewhat greater role at later stages. Fungal communities appeared more sensitive to host-specific selective environments. Seasonal effects on the relative contribution of assembly processes were negligible.

## Discussion

Despite pronounced changes in soil nutrient availability and ecosystem properties along the studied chronosequence [61], foliar endophyte communities exhibited relatively modest shifts across the ecosystem development gradient. Host plant identity consistently explained more variation in foliar endophyte composition than did ecosystem development stage or season. This pattern was evident for both bacterial and fungal communities, and across multiple analytical approaches. Although ecosystem age strongly influenced soil properties and belowground microbial communities at the experimental sites, these findings suggest that, aboveground, host-specific filtering mechanisms, such as the leaf stoichiometry analyzed here, are likely to exert stronger control over endophyte assembly than the broader successional context. Foliar tissues represent comparatively transient and spatially isolated habitats that are partially decoupled from belowground successional processes [2]. The phyllosphere is continuously exposed to airborne microbial influx, which may homogenize communities across sites and dampen the influence of local ecosystem characteristics [62]. In addition, the relatively short lifespan and continual turnover of leaves may constrain the accumulation of long-term successional legacies within aboveground microbial assemblages [2, 5, 63]. Thus, while ecosystem development is known to restructure below-ground environments, its imprint on foliar endophytes appears comparatively weak.

Because the successional stage was represented by spatially distinct sites within a relatively small geographic area, we cannot fully disentangle the effects of ecosystem development from unmeasured site-specific factors. As is the case in most space-for-time substitution studies, the ecosystem development stage and sampling site are inherently joined in the design. However, the four sites sampled here share common parent material, regional climate, and species pool, and the observed differences in soil C, N, and microbial biomass are intrinsic components of ecosystem development along the chronosequence rather than independent confounding factors. Our results, therefore, described patterns associated with ecosystem developmental stage under these shared regional conditions, rather than temporal causality. In addition, null model analyses indicated that community turnover was largely consistent with stochastic assembly processes, with ecological drift accounting for the majority of the stochastic signal. Ecological drift is a central concept in community ecology and refers to random changes in organism abundance within a community over time [64]. It has been predicted to play a critical role in shaping the structure of microbial communities, especially under conditions of weak selection and small local community size [65]. The open and dynamic nature of the phyllosphere—characterized by the continuous presence of airborne microbes, fluctuating microclimatic conditions, and short microbial generation times—likely favors such stochastic assembly dynamics. The high species turnover rates observed here are also consistent with a few published studies on microbial community assembly, in which ecological drift dominance, coupled with weak selection or small local population sizes, increased community variation and species turnover [47, 66].

Although the taxonomic composition varied among hosts and across the four ecosystem stages, the proportional representation of inferred functional genes remained relatively stable. We consistently detected genera containing members known to harbor genes associated with nitrogen fixation, methylotrophy, nitrification, and denitrification across hosts and successional stages. This decoupling of taxonomic turnover from predicted ecological function suggests a degree of functional redundancy within foliar endophyte communities. Foliar N_2_-fixation has been confirmed previously in coniferous tree needles [67], while methylotrophs are a commonly reported group of endophytic bacteria across many studies [61]. Methylotrophic bacteria use single-C compounds such as methanol as their sole source of C and energy. Methanol is the second most abundant volatile organic compound in the atmosphere, with the majority of it produced as a metabolic by-product during plant growth [68]. Molecular N is also readily available in the aerated intercellular spaces of plants. Therefore, the metabolic capacity to fix both C and N from their gaseous forms appears to be a key ecological trait for bacterial endophytes in the foliar endosphere. Apart from methanol, plants have been suggested to generate substantial amounts of nitrous oxide (N_2_O) [69, 70]. The mechanism, however, is not well understood, as plant cells cannot form N_2_O by reducing nitric oxide (NO)—a step commonly performed by denitrifying or co-denitrifying bacteria and fungi. In accord with the “endophyte-plant co-denitrification” hypothesis [71], microbial genera with predicted denitrification potential were highly prevalent in our dataset, with high relative abundances across all studied plant host species throughout the growing season. Future studies integrating functional gene quantification and/or metagenomics will help clarify the extent to which functional redundancy contributes to the conservation of these or other microbial functions in the phyllosphere.

Co-occurrence networks differed markedly among hosts, indicating that plant identity shapes not only taxonomic composition, but also potential interaction architecture. The increased detection of negative associations from spring to summer suggests that competitive or niche-differentiation processes may also play important roles within the endosphere. These findings further suggest that as the endosphere community develops and colonization space becomes limited, fungal taxa may assume a greater ecological role, potentially reflecting increased niche differentiation and fungi's capacity to structure microbial interactions within plant tissues.

Similar to previously published results [72] the structure of microbiomes colonizing the leaves of all four studied plant hosts underwent dynamic shifts during the growing season. In spring, the endophytic communities exhibited high similarity, except for the non-deciduous *P. abies*, whose needles likely harbored overwintering endophyte taxa. This suggests that the environmental microbial inoculum capable of colonizing the foliar endosphere of sterile spring leaves is common to all studied host species. The overlap was smaller in summer, and by autumn, the foliar microbiomes of the plant hosts studied were highly dissimilar. At the same time, microbial turnover rates decreased from summer to autumn, and the autumn cross-domain co-occurrence networks were dominated by Ascomycota and Basidiomycota fungi, indicating the establishment of plant host species-specific fungal pathogens and saprotrophs in the foliar endosphere [72].

Together, our findings indicate that foliar endophyte communities across a primary successional gradient are structured primarily by plant host filtering and stochastic processes rather than by deterministic effects of ecosystem age. Despite substantial taxonomic turnover, functional potential appears to be buffered by redundancy, suggesting resilience in aboveground microbial functions during ecosystem development. Based on this study and others [18, 61], we suggest that our predictive understanding of plant-associated microbiomes will benefit from studies that simultaneously consider host and microbial traits, environmental context, temporal dynamics, and stochastic processes. Incorporating these factors together can help identify generalizable patterns and mechanisms governing microbial colonization of plant hosts.

## Supplementary Information

Below is the link to the electronic supplementary material.

Supplementary Material 1: Fig. S1. Map showing the study area at four unreclaimed post-lignite mining sites near the city of Sokolov, Czech Republic: Location I (10 years since abandonment), Location II (20 years), Location III (30 years), and Location IV (54 years). Panels A–D show vegetation development at each site. Fig. S2. Relationships among environmental variables (host plant species, seasonal age, and successional gradient) and leaf tissue stoichiometry are visualized in a redundancy analysis (RDA) biplot. The four plant species include (*Calamagrostis epigejos* (Cala_epi), *Picea abies* (Pice_abi), *Salix caprea* (Sali_cap), and *Tussilago farfara* (Tuss_far)), across four successional stages (I, II, III, and IV), along a single growing season (Spring, Summer, and Autumn). Fig. S3. Total number and proportion (%) of bacterial genera shared among different host plant species [*Calamagrostis epigejo*s (CE), *Picea abies* (PA), *Salix caprea* (SC), and *Tussilago farfara* (TF)] across the ecological succession gradient (Locations I, II, III, and IV) during a single growing season (spring, summer, and autumn). Fig. S4. Total number and proportion (%) of fungal genera shared among different host plant species [*Calamagrostis epigejos* (CE), *Picea abies* (PA), *Salix caprea* (SC), and *Tussilago farfara* (TF)] across the ecological succession gradient (Locations I, II, III, and IV) during the plant growth period (spring, summer, and autumn). Fig. S5. Diversity indices for foliar endophyte communities obtained from experimental sites differing in successional age (I, II, III, and IV), during a single growing season (spring, summer, and autumn). Bacterial (A) and fungal (B) richness (Chao1), and bacterial (C) and fungal (D) diversity (Shannon–Wiener) are shown. Values from the host plant species growing at the same location and sampled at the same time were pooled together for the analysis. Different letters indicate a significant difference in the indices for the same season along the successional stage. Fig. S6. Heatmap showing Kendall correlation coefficients for the bacterial (S6a, S6b, and S6c) and fungal (S6d, S6e, and S6f) taxa, and the foliar nutrient content (carbon, C; nitrogen, N; phosphorous, P), including molar ratios (carbon:nitrogen, C:N; carbon:phosphorus, C:P; and nitrogen:phosphorus, N:P) in the four studied host plants (*Calamagrostis epigejos*, *Picea abies*, *Salix caprea*, and *Tussilago farfara*, Figures S6a and S6d), at the four sampled sites (I, II, III, and IV—Figures S6b and S6e), at the three different sampling points (spring, summer, autumn, Figures S6c and S6f). The p values significant at the 0.001, 0.01, and 0.05 levels are represented by “***”, “**”, “*”respectively. Fig. S7. Linear discriminant analysis (LDA) effect size (LEfSe) displaying significant indicator taxa colonizing the foliar endosphere of the four studied host plants (A), during the growing season (B), with the taxonomic classification at the genus level. Bar graphs represent the LDA score. Fig. S8. Zi-Pi plot, indicating the distribution of nodes based on their topological roles in cross-domain co-occurrence networks constructed for the microbiomes colonizing the four plant host species (*Calamagrostis epigejos*, *Picea abies*, *Salix caprea*, and *Tussilago farfara*), growing at different stages of ecological succession (I, II, III, and IV) during a single growing season (spring, summer, and autumn). Each symbol represents a genus—circles for bacteria and squares for fungi—and symbol size indicates relative abundance. Plant identity is represented by different colors. Nodes were categorized into four functional types based on their topological characteristics. Connectors: Nodes with high connectivity within two modules (Zi > 2.5 and Pi < 0.6). Module hubs: Nodes with high connectivity between two modules (Zi < 2.5 and Pi > 0.62). Network hubs: Nodes with high connectivity (Zi > 2.5 and Pi > 0.62). Peripherals: Nodes that do not exhibit high connectivity (Zi < 2.5 and Pi < 0.62) either within or between modules.

Supplementary Material 2

Supplementary Material 3

Supplementary Material 4

Supplementary Material 5

Supplementary Material 6

Supplementary Material 7

Supplementary Material 8

Supplementary Material 9

Supplementary Material 10

## Data Availability

The raw sequences have been deposited in the NCBI Sequence Read Archive under the BioProject accession numbers PRJNA894138 (16S) and PRJNA894357 (ITS). All other relevant supporting data is avaible as supplementary information.
