# Supplementary material for: Ecological drift and host filtering jointly structure foliar endophytes during ecosystem development
Source: Environ Microbiome. 2026 May 8;21:83. doi: 10.1186/s40793-026-00906-7 (PMC13321508; doi:10.1186/s40793-026-00906-7)
Supplement: Supplementary file 1 — Supplementary Material 1: Fig. S1. Map showing the study area at four unreclaimed post-lignite mining sites near the city of Sokolov, Czech Republic: Location I (10 years since abandonment), Location II (20 years), Location III (30 years), and Location IV (54 years). Panels A–D show vegetation development at each site. Fig. S2. Relationships among environmental variables (host plant species, seasonal age, and successional gradient) and leaf tissue stoichiometry are visualized in a redundancy analysis (RDA) biplot. The four plant species include (Calamagrostis epigejos (Cala_epi), Picea abies (Pice_abi), Salix caprea (Sali_cap), and Tussilago farfara (Tuss_far)), across four successional stages (I, II, III, and IV), along a single growing season (Spring, Summer, and Autumn). Fig. S3. Total number and proportion (%) of bacterial genera shared among different host plant species [Calamagrostis epigejos (CE), Picea abies (PA), Salix caprea (SC), and Tussilago farfara (TF)] across the ecological succession gradient (Locations I, II, III, and IV) during a single growing season (spring, summer, and autumn). Fig. S4. Total number and proportion (%) of fungal genera shared among different host plant species [Calamagrostis epigejos (CE), Picea abies (PA), Salix caprea (SC), and Tussilago farfara (TF)] across the ecological succession gradient (Locations I, II, III, and IV) during the plant growth period (spring, summer, and autumn). Fig. S5. Diversity indices for foliar endophyte communities obtained from experimental sites differing in successional age (I, II, III, and IV), during a single growing season (spring, summer, and autumn). Bacterial (A) and fungal (B) richness (Chao1), and bacterial (C) and fungal (D) diversity (Shannon–Wiener) are shown. Values from the host plant species growing at the same location and sampled at the same time were pooled together for the analysis. Different letters indicate a significant difference in the indices for the same season along the s [file 40793_2026_906_MOESM1_ESM.pdf]

Figure S1

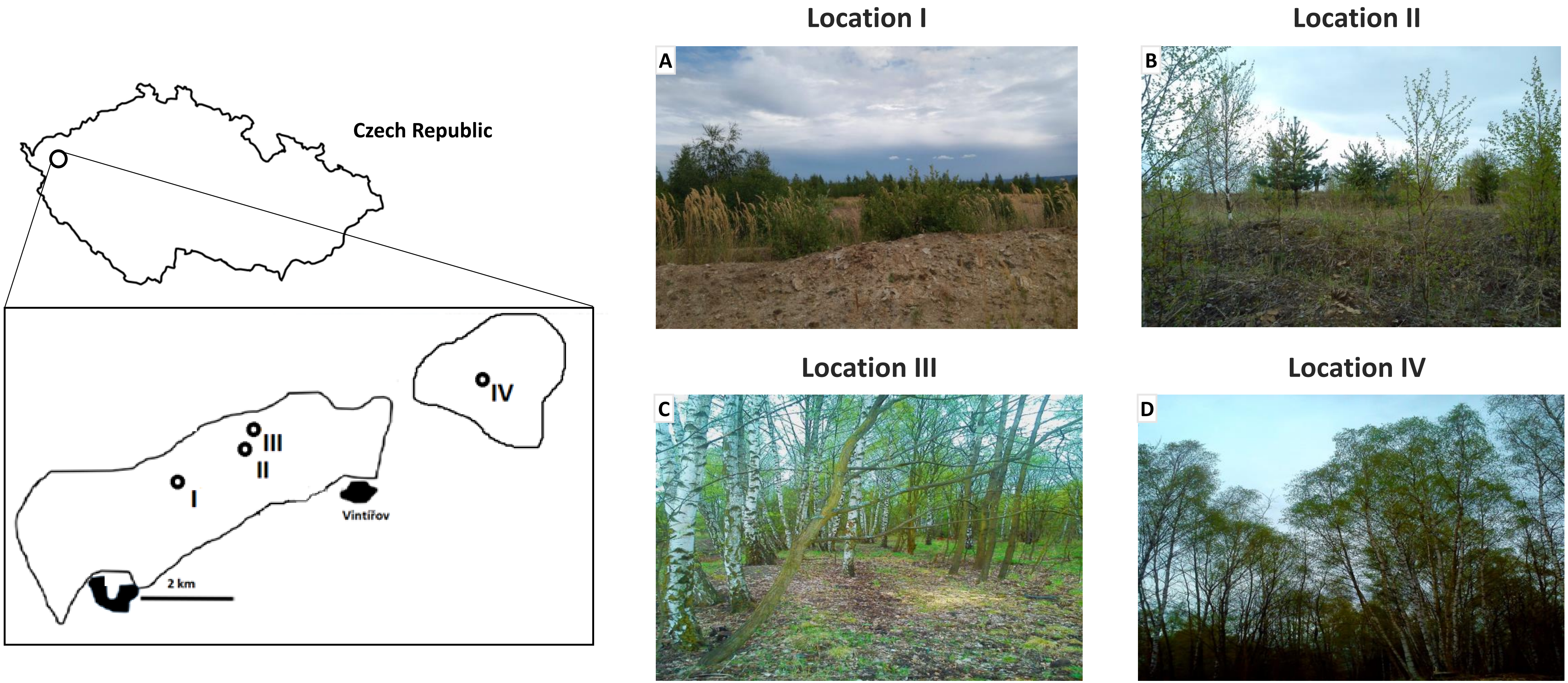
Czech Republic
IV
III
II
I
Vintířov
2 km
Location I
A
Location II
B
Location III
C
Location IV
D

Figure S2

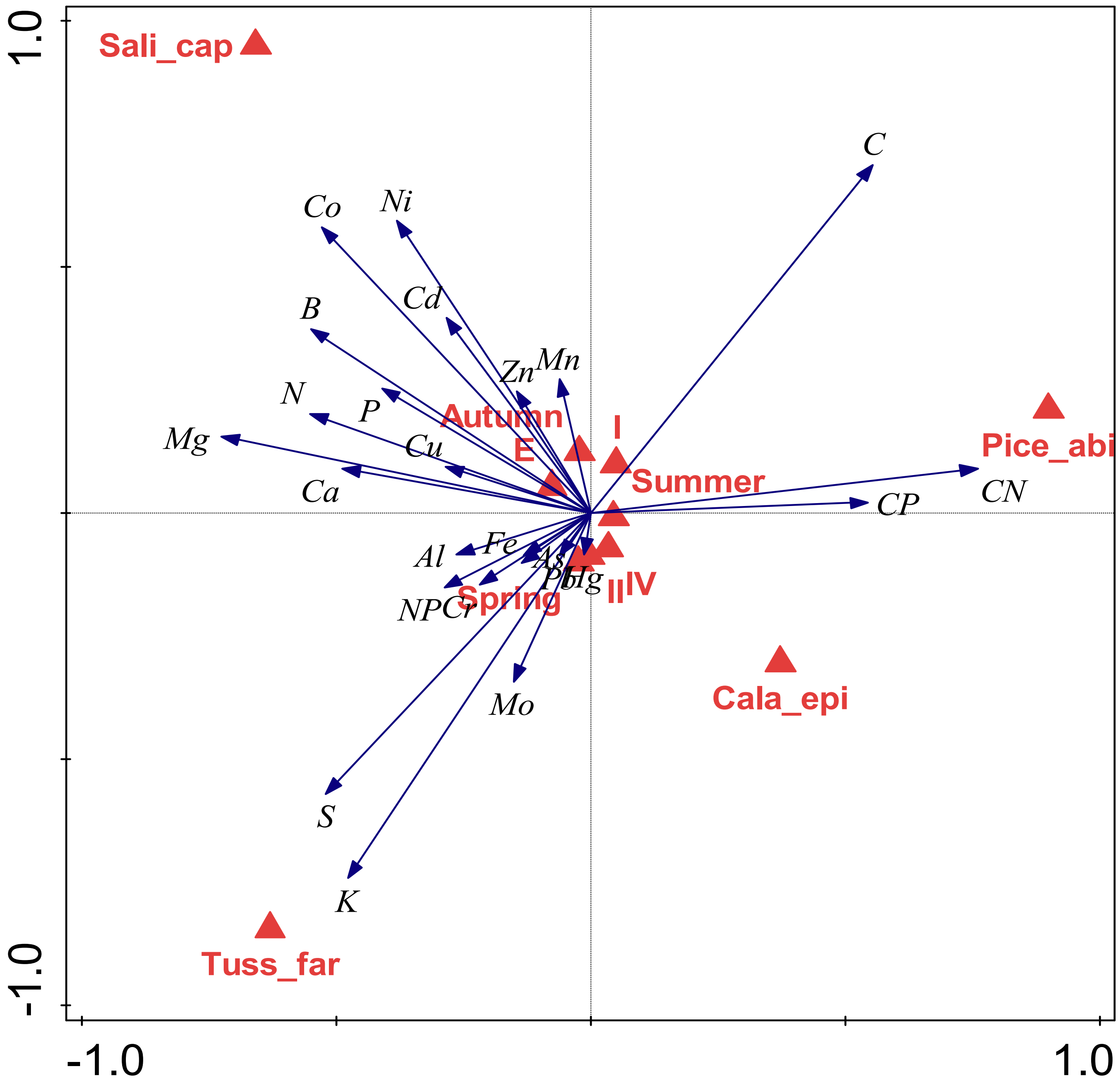

1.0
-1.0
-1.0
1.0
Sali_cap
Pice_abi
Cala_epi
Tuss_far
Autumn
Summer
Spring
E
I
II
IV
C
Co
Ni
Cd
B
Zn
Mn
N
P
Mg
Cu
Ca
CP
CN
Al
Fe
As
Pb
Hg
NPCr
Mo
S
K

Figure S3

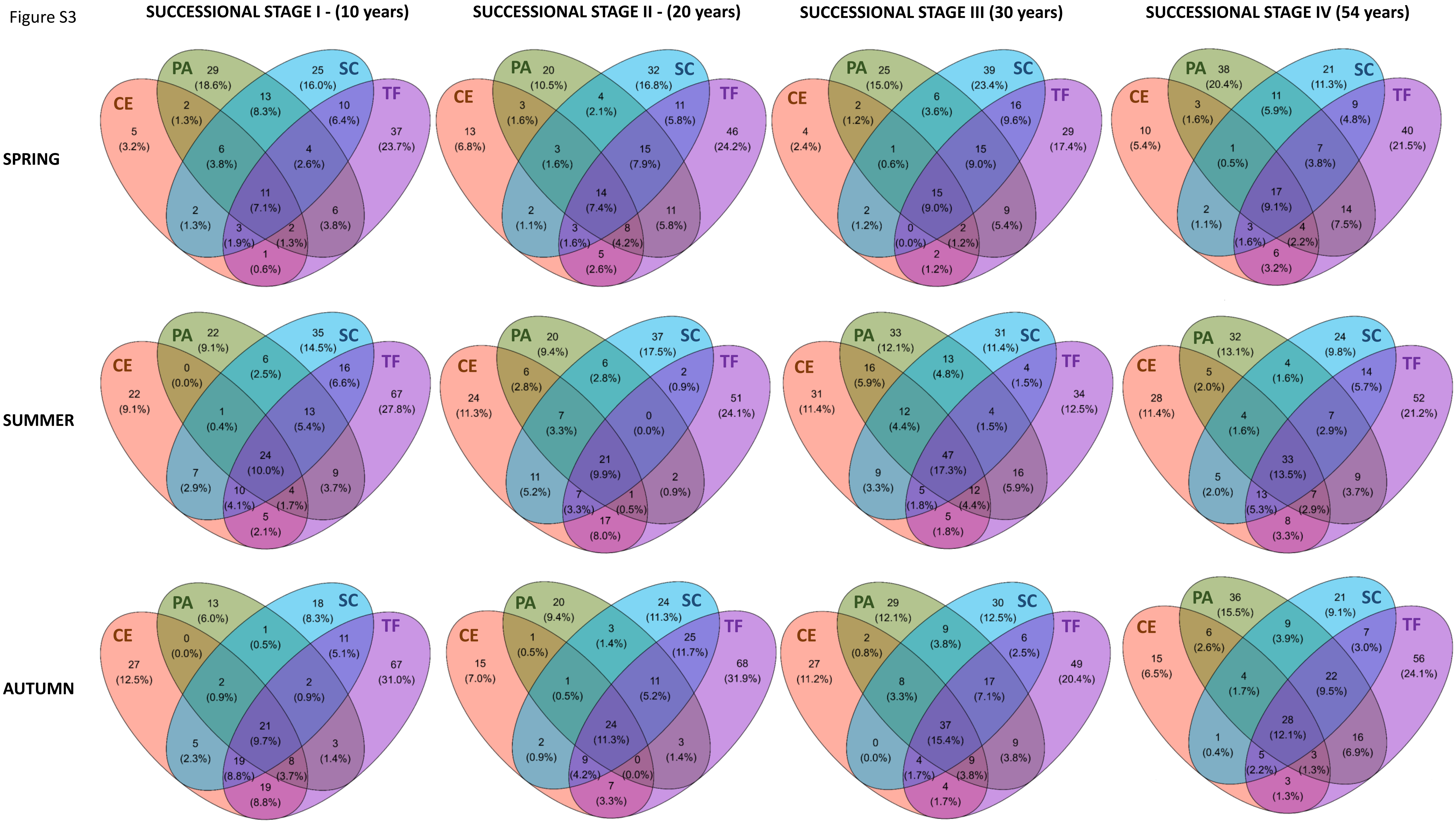
SUCCESSIONAL STAGE I - (10 years)
SUCCESSIONAL STAGE II - (20 years)
SUCCESSIONAL STAGE III (30 years)
SUCCESSIONAL STAGE IV (54 years)
SPRING
CE PA SC TF
29 (18.6%)
25 (16.0%)
5 (3.2%)
37 (23.7%)
2 (1.3%)
13 (8.3%)
10 (6.4%)
6 (3.8%)
4 (2.6%)
11 (7.1%)
2 (1.3%)
6 (3.8%)
3 (1.9%)
2 (1.3%)
1 (0.6%)
CE PA SC TF
20 (10.5%)
32 (16.8%)
13 (6.8%)
46 (24.2%)
3 (1.6%)
4 (2.1%)
11 (5.8%)
3 (1.6%)
15 (7.9%)
14 (7.4%)
2 (1.1%)
11 (5.8%)
3 (1.6%)
8 (4.2%)
5 (2.6%)
CE PA SC TF
25 (15.0%)
39 (23.4%)
4 (2.4%)
29 (17.4%)
2 (1.2%)
6 (3.6%)
16 (9.6%)
1 (0.6%)
15 (9.0%)
15 (9.0%)
2 (1.2%)
9 (5.4%)
0 (0.0%)
2 (1.2%)
2 (1.2%)
CE PA SC TF
38 (20.4%)
21 (11.3%)
10 (5.4%)
40 (21.5%)
3 (1.6%)
11 (5.9%)
9 (4.8%)
1 (0.5%)
7 (3.8%)
17 (9.1%)
2 (1.1%)
14 (7.5%)
3 (1.6%)
4 (2.2%)
6 (3.2%)
SUMMER
CE PA SC TF
22 (9.1%)
35 (14.5%)
22 (9.1%)
67 (27.8%)
0 (0.0%)
6 (2.5%)
16 (6.6%)
1 (0.4%)
13 (5.4%)
24 (10.0%)
7 (2.9%)
9 (3.7%)
10 (4.1%)
4 (1.7%)
5 (2.1%)
CE PA SC TF
20 (9.4%)
37 (17.5%)
24 (11.3%)
51 (24.1%)
6 (2.8%)
6 (2.8%)
2 (0.9%)
7 (3.3%)
0 (0.0%)
21 (9.9%)
11 (5.2%)
2 (0.9%)
7 (3.3%)
1 (0.5%)
17 (8.0%)
CE PA SC TF
33 (12.1%)
31 (11.4%)
31 (11.4%)
34 (12.5%)
16 (5.9%)
13 (4.8%)
4 (1.5%)
12 (4.4%)
4 (1.5%)
47 (17.3%)
9 (3.3%)
16 (5.9%)
5 (1.8%)
12 (4.4%)
5 (1.8%)
CE PA SC TF
32 (13.1%)
24 (9.8%)
28 (11.4%)
52 (21.2%)
5 (2.0%)
4 (1.6%)
14 (5.7%)
4 (1.6%)
7 (2.9%)
33 (13.5%)
5 (2.0%)
9 (3.7%)
13 (5.3%)
7 (2.9%)
8 (3.3%)
AUTUMN
CE PA SC TF
13 (6.0%)
18 (8.3%)
27 (12.5%)
67 (31.0%)
0 (0.0%)
1 (0.5%)
11 (5.1%)
2 (0.9%)
2 (0.9%)
21 (9.7%)
5 (2.3%)
3 (1.4%)
19 (8.8%)
8 (3.7%)
19 (8.8%)
CE PA SC TF
20 (9.4%)
24 (11.3%)
15 (7.0%)
68 (31.9%)
1 (0.5%)
3 (1.4%)
25 (11.7%)
1 (0.5%)
11 (5.2%)
24 (11.3%)
2 (0.9%)
3 (1.4%)
9 (4.2%)
0 (0.0%)
7 (3.3%)
CE PA SC TF
29 (12.1%)
30 (12.5%)
27 (11.2%)
49 (20.4%)
2 (0.8%)
9 (3.8%)
6 (2.5%)
8 (3.3%)
17 (7.1%)
37 (15.4%)
0 (0.0%)
9 (3.8%)
4 (1.7%)
9 (3.8%)
4 (1.7%)
CE PA SC TF
36 (15.5%)
21 (9.1%)
15 (6.5%)
56 (24.1%)
6 (2.6%)
9 (3.9%)
7 (3.0%)
4 (1.7%)
22 (9.5%)
28 (12.1%)
1 (0.4%)
16 (6.9%)
5 (2.2%)
3 (1.3%)
3 (1.3%)

Figure S4

SUCCESSIONAL STAGE I - (10 years) | SUCCESSIONAL STAGE II - (20 years) | SUCCESSIONAL STAGE III (30 years) | SUCCESSIONAL STAGE IV (54 years)

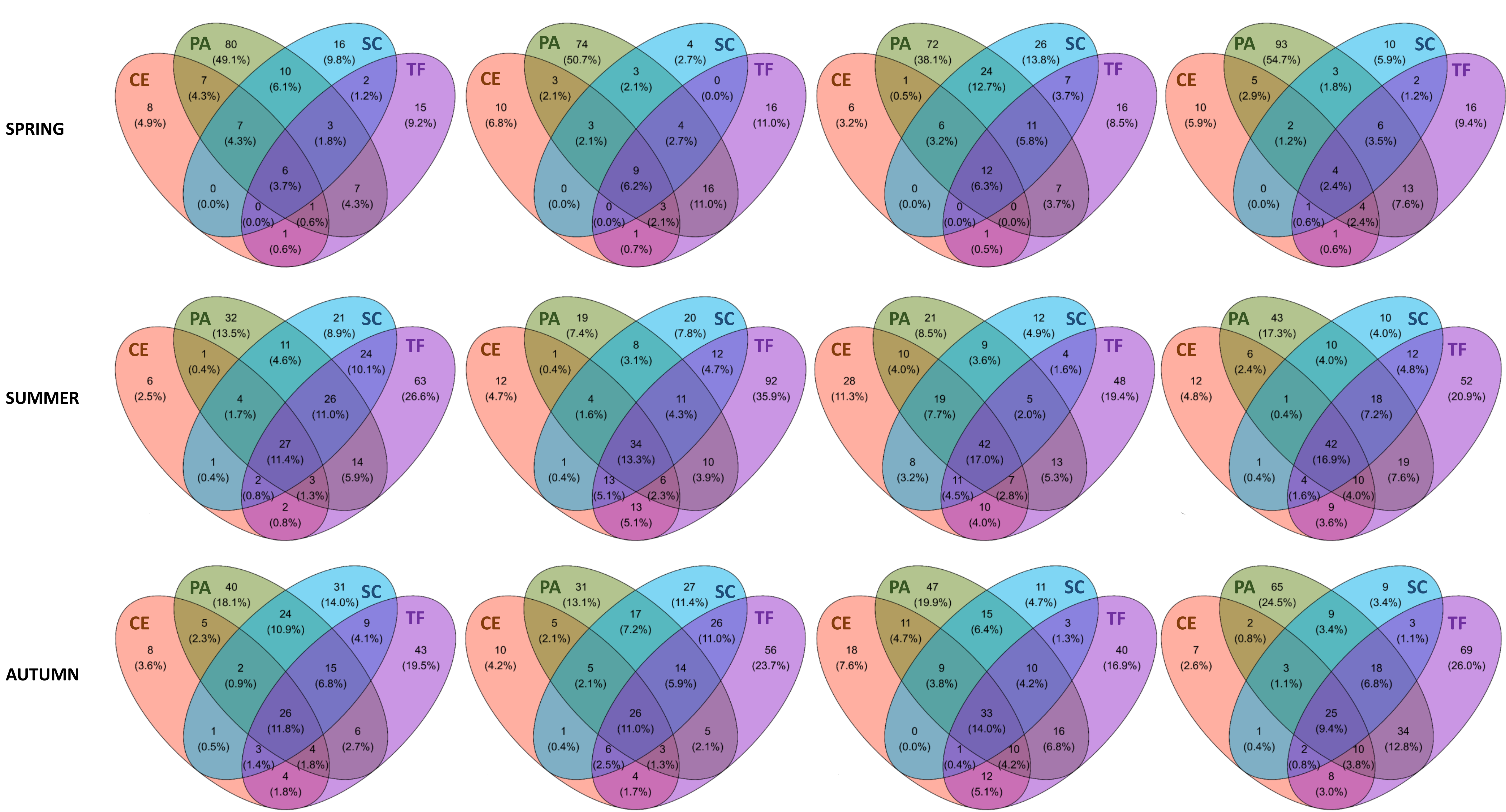

Figure S5

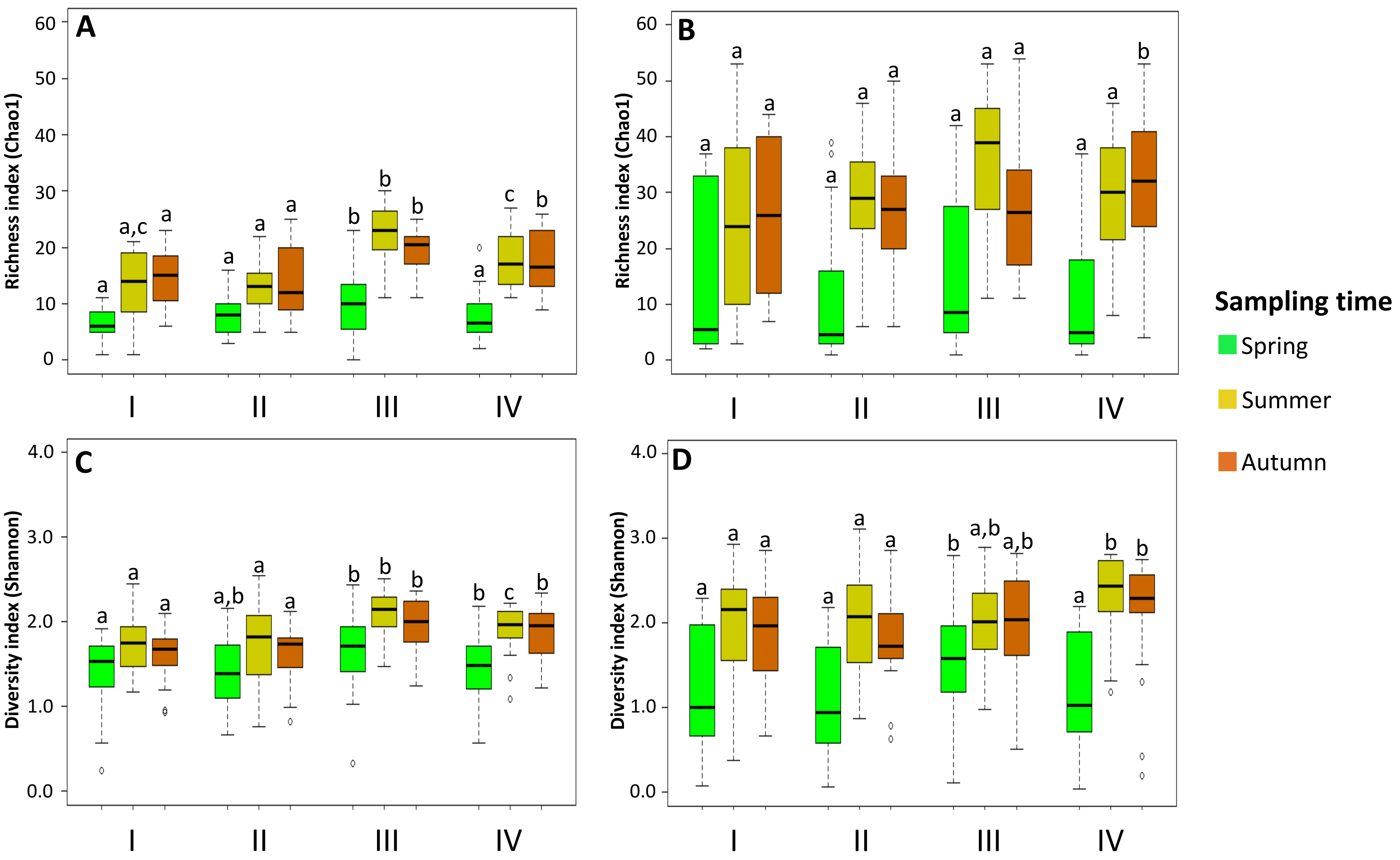
A
B
C
D
Richness index (Chao1)
Diversity index (Shannon)
I
II
III
IV
Sampling time
Spring
Summer
Autumn

Figure S6a

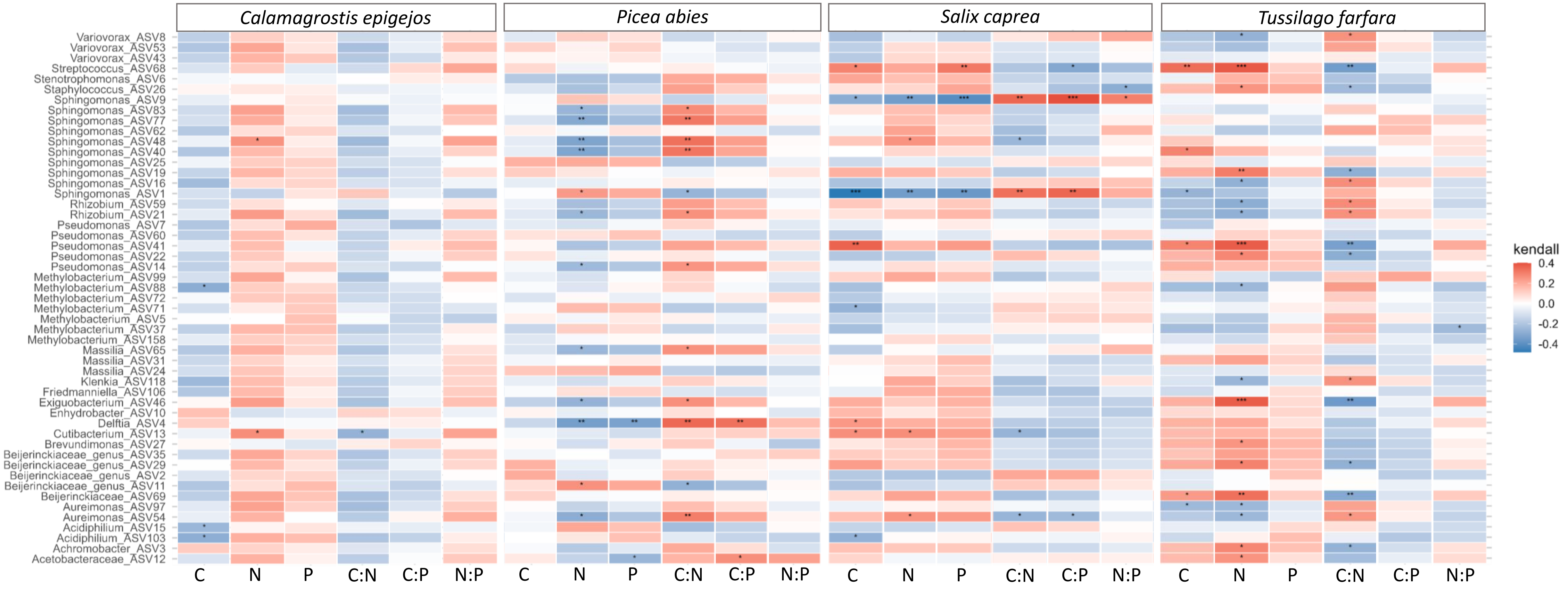

Calamagrostis epigejos
Picea abies
Salix caprea
Tussilago farfara
Variovorax_ASV8
Variovorax_ASV53
Variovorax_ASV43
Streptococcus_ASV68
Stenotrophomonas_ASV6
Staphylococcus_ASV26
Sphingomonas_ASV9
Sphingomonas_ASV83
Sphingomonas_ASV77
Sphingomonas_ASV62
Sphingomonas_ASV48
Sphingomonas_ASV40
Sphingomonas_ASV25
Sphingomonas_ASV19
Sphingomonas_ASV16
Sphingomonas_ASV1
Rhizobium_ASV59
Rhizobium_ASV21
Pseudomonas_ASV7
Pseudomonas_ASV60
Pseudomonas_ASV41
Pseudomonas_ASV22
Pseudomonas_ASV14
Methylobacterium_ASV99
Methylobacterium_ASV88
Methylobacterium_ASV72
Methylobacterium_ASV71
Methylobacterium_ASV5
Methylobacterium_ASV37
Methylobacterium_ASV158
Massilia_ASV65
Massilia_ASV31
Massilia_ASV24
Klenkia_ASV118
Friedmanniella_ASV106
Exiguobacterium_ASV46
Enhydrobacter_ASV10
Delftia_ASV4
Cutibacterium_ASV13
Brevundimonas_ASV27
Beijerinckiaceae_genus_ASV35
Beijerinckiaceae_genus_ASV29
Beijerinckiaceae_genus_ASV2
Beijerinckiaceae_genus_ASV11
Beijerinckiaceae_ASV69
Aureimonas_ASV97
Aureimonas_ASV54
Acidiphilium_ASV15
Acidiphilium_ASV103
Achromobacter_ASV3
Acetobacteraceae_ASV12
C N P C:N C:P N:P
C N P C:N C:P N:P
C N P C:N C:P N:P
C N P C:N C:P N:P
kendall
0.4
0.2
0.0
-0.2
-0.4

Figure S6b

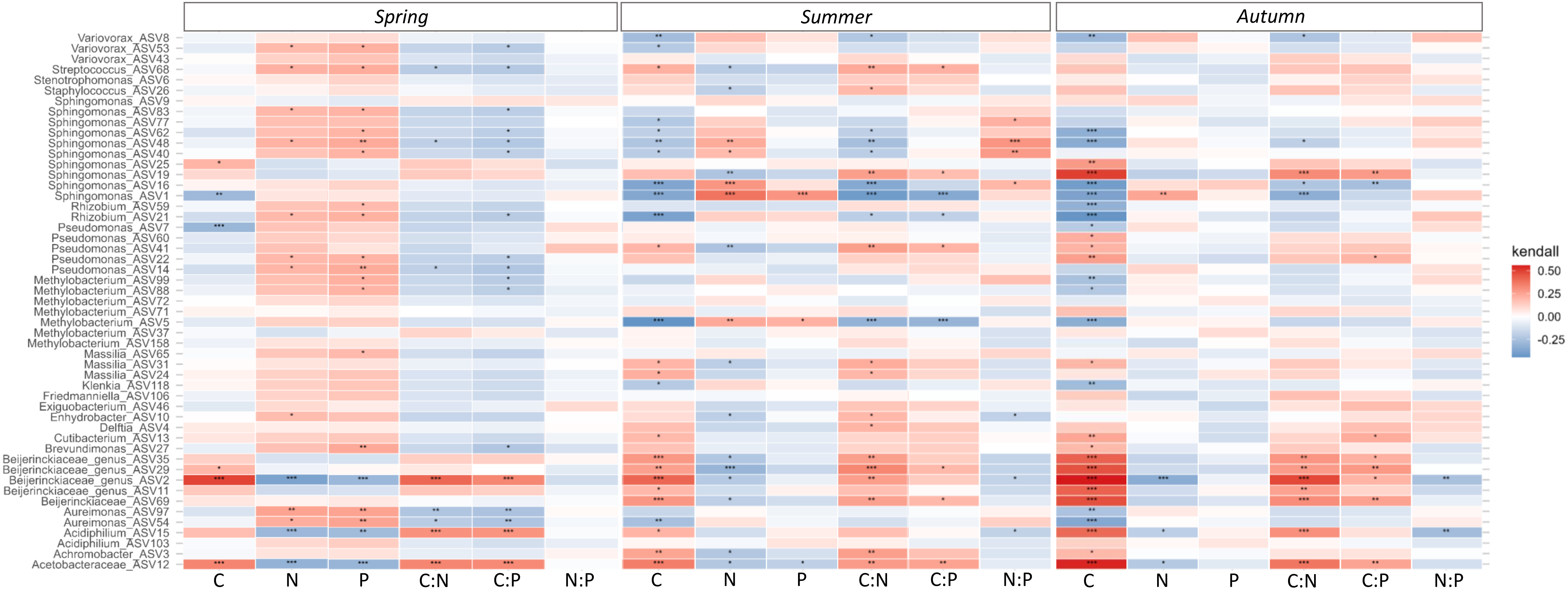
Spring
Summer
Autumn
Variovorax_ASV8
Variovorax_ASV53
Variovorax_ASV43
Streptococcus_ASV68
Stenotrophomonas_ASV6
Staphylococcus_ASV26
Sphingomonas_ASV9
Sphingomonas_ASV83
Sphingomonas_ASV77
Sphingomonas_ASV62
Sphingomonas_ASV48
Sphingomonas_ASV40
Sphingomonas_ASV25
Sphingomonas_ASV19
Sphingomonas_ASV16
Sphingomonas_ASV1
Rhizobium_ASV59
Rhizobium_ASV21
Pseudomonas_ASV7
Pseudomonas_ASV60
Pseudomonas_ASV41
Pseudomonas_ASV22
Pseudomonas_ASV14
Methylobacterium_ASV99
Methylobacterium_ASV88
Methylobacterium_ASV72
Methylobacterium_ASV71
Methylobacterium_ASV5
Methylobacterium_ASV37
Methylobacterium_ASV158
Massilia_ASV65
Massilia_ASV31
Massilia_ASV24
Klenkia_ASV118
Friedmanniella_ASV106
Exiguobacterium_ASV46
Enhydrobacter_ASV10
Delftia_ASV4
Cutibacterium_ASV13
Brevundimonas_ASV27
Beijerinckiaceae_genus_ASV35
Beijerinckiaceae_genus_ASV29
Beijerinckiaceae_genus_ASV2
Beijerinckiaceae_genus_ASV11
Beijerinckiaceae_ASV69
Aureimonas_ASV97
Aureimonas_ASV54
Acidiphilium_ASV15
Acidiphilium_ASV103
Achromobacter_ASV3
Acetobacteraceae_ASV12
C
N
P
C:N
C:P
N:P
kendall
0.50
0.25
0.00
-0.25

Figure S6c

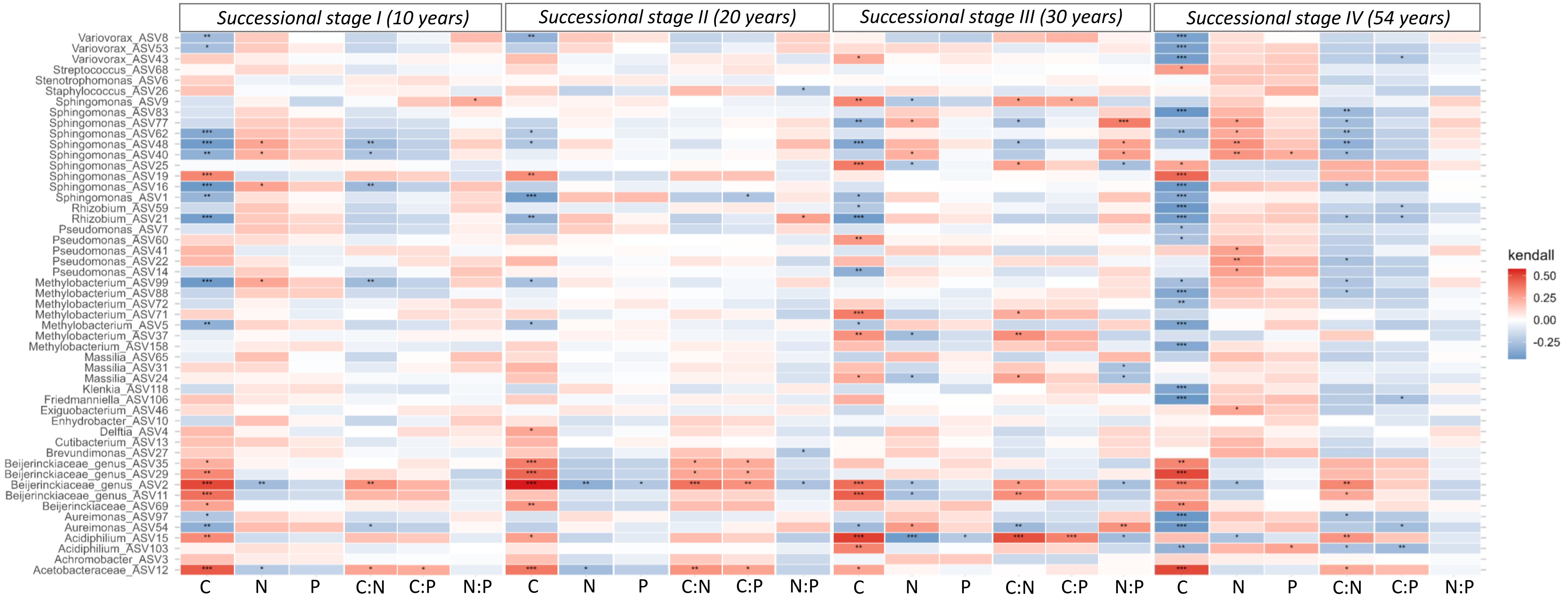

Successional stage I (10 years)
Successional stage II (20 years)
Successional stage III (30 years)
Successional stage IV (54 years)
Variovorax_ASV8
Variovorax_ASV53
Variovorax_ASV43
Streptococcus_ASV68
Stenotrophomonas_ASV6
Staphylococcus_ASV26
Sphingomonas_ASV9
Sphingomonas_ASV83
Sphingomonas_ASV77
Sphingomonas_ASV62
Sphingomonas_ASV48
Sphingomonas_ASV40
Sphingomonas_ASV25
Sphingomonas_ASV19
Sphingomonas_ASV16
Sphingomonas_ASV1
Rhizobium_ASV59
Rhizobium_ASV21
Pseudomonas_ASV7
Pseudomonas_ASV60
Pseudomonas_ASV41
Pseudomonas_ASV22
Pseudomonas_ASV14
Methylobacterium_ASV99
Methylobacterium_ASV88
Methylobacterium_ASV72
Methylobacterium_ASV71
Methylobacterium_ASV5
Methylobacterium_ASV37
Methylobacterium_ASV158
Massilia_ASV65
Massilia_ASV31
Massilia_ASV24
Klenkia_ASV118
Friedmanniella_ASV106
Exiguobacterium_ASV46
Enhydrobacter_ASV10
Delftia_ASV4
Cutibacterium_ASV13
Brevundimonas_ASV27
Beijerinckiaceae_genus_ASV35
Beijerinckiaceae_genus_ASV29
Beijerinckiaceae_genus_ASV2
Beijerinckiaceae_genus_ASV11
Beijerinckiaceae_ASV69
Aureimonas_ASV97
Aureimonas_ASV54
Acidiphilium_ASV15
Acidiphilium_ASV103
Achromobacter_ASV3
Acetobacteraceae_ASV12
C N P C:N C:P N:P
C N P C:N C:P N:P
C N P C:N C:P N:P
C N P C:N C:P N:P
kendall
0.50
0.25
0.00
-0.25

Figure S6d

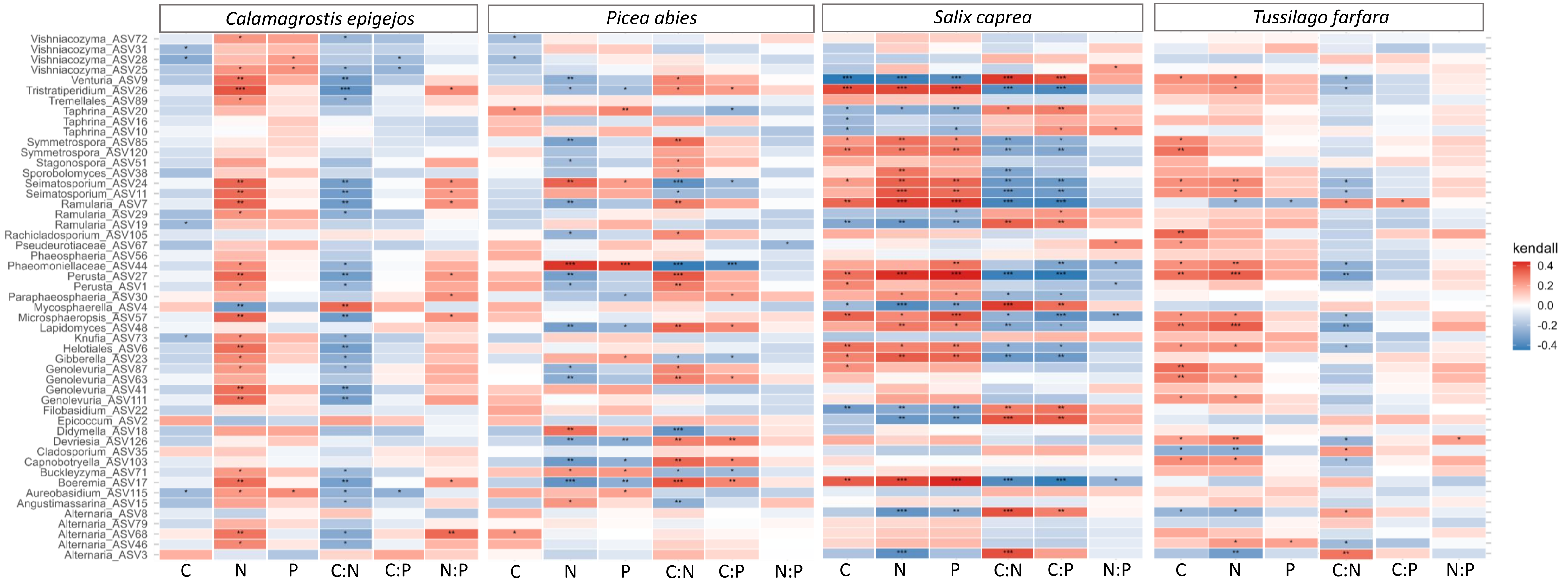
Calamagrostis epigejos
Picea abies
Salix caprea
Tussilago farfara
Vishniacozyma_ASV72
Vishniacozyma_ASV31
Vishniacozyma_ASV28
Vishniacozyma_ASV25
Venturia_ASV9
Tristratiperidium_ASV26
Tremellales_ASV89
Taphrina_ASV20
Taphrina_ASV16
Taphrina_ASV10
Symmetrospora_ASV85
Symmetrospora_ASV120
Stagonospora_ASV51
Sporobolomyces_ASV38
Seimatosporium_ASV24
Seimatosporium_ASV11
Ramularia_ASV7
Ramularia_ASV29
Ramularia_ASV19
Rachicladosporium_ASV105
Pseudeurotiaceae_ASV67
Phaeosphaeria_ASV56
Phaeomoniellaceae_ASV44
Perusta_ASV27
Perusta_ASV1
Paraphaeosphaeria_ASV30
Mycosphaerella_ASV4
Microsphaeropsis_ASV57
Lapidomyces_ASV48
Knufia_ASV73
Helotiales_ASV6
Gibberella_ASV23
Genolevuria_ASV87
Genolevuria_ASV63
Genolevuria_ASV41
Genolevuria_ASV111
Filobasidium_ASV22
Epicoccum_ASV2
Didymella_ASV18
Devriesia_ASV126
Cladosporium_ASV35
Capnobotryella_ASV103
Buckleyzyma_ASV71
Boeremia_ASV17
Aureobasidium_ASV115
Angustimassarina_ASV15
Alternaria_ASV8
Alternaria_ASV79
Alternaria_ASV68
Alternaria_ASV46
Alternaria_ASV3
C N P C:N C:P N:P
C N P C:N C:P N:P
C N P C:N C:P N:P
C N P C:N C:P N:P
kendall
0.4
0.2
0.0
-0.2
-0.4

Figure S6e

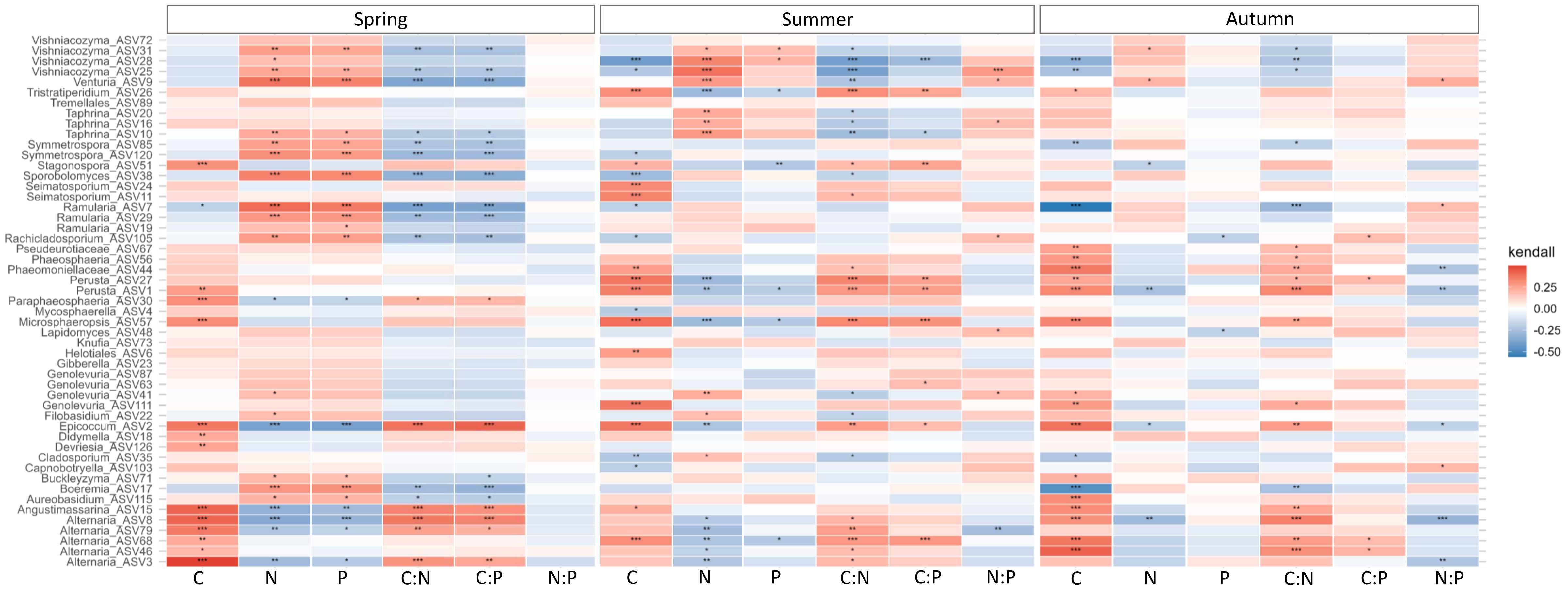
Spring
Summer
Autumn
Vishniacozyma_ASV72
Vishniacozyma_ASV31
Vishniacozyma_ASV28
Vishniacozyma_ASV25
Venturia_ASV9
Tristratiperidium_ASV26
Tremellales_ASV89
Taphrina_ASV20
Taphrina_ASV16
Taphrina_ASV10
Symmetrospora_ASV85
Symmetrospora_ASV120
Stagonospora_ASV51
Sporobolomyces_ASV38
Seimatosporium_ASV24
Seimatosporium_ASV11
Ramularia_ASV7
Ramularia_ASV29
Ramularia_ASV19
Rachicladosporium_ASV105
Pseudeurotiaceae_ASV67
Phaeosphaeria_ASV56
Phaeomoniellaceae_ASV44
Perusta_ASV27
Perusta_ASV1
Paraphaeosphaeria_ASV30
Mycosphaerella_ASV4
Microsphaeropsis_ASV57
Lapidomyces_ASV48
Knufia_ASV73
Helotiales_ASV6
Gibberella_ASV23
Genolevuria_ASV87
Genolevuria_ASV63
Genolevuria_ASV41
Genolevuria_ASV111
Filobasidium_ASV22
Epicoccum_ASV2
Didymella_ASV18
Devriesia_ASV126
Cladosporium_ASV35
Capnobotryella_ASV103
Buckleyzyma_ASV71
Boeremia_ASV17
Aureobasidium_ASV115
Angustimassarina_ASV15
Alternaria_ASV8
Alternaria_ASV79
Alternaria_ASV68
Alternaria_ASV46
Alternaria_ASV3
C
N
P
C:N
C:P
N:P
kendall
0.25
0.00
-0.25
-0.50

Figure S6f

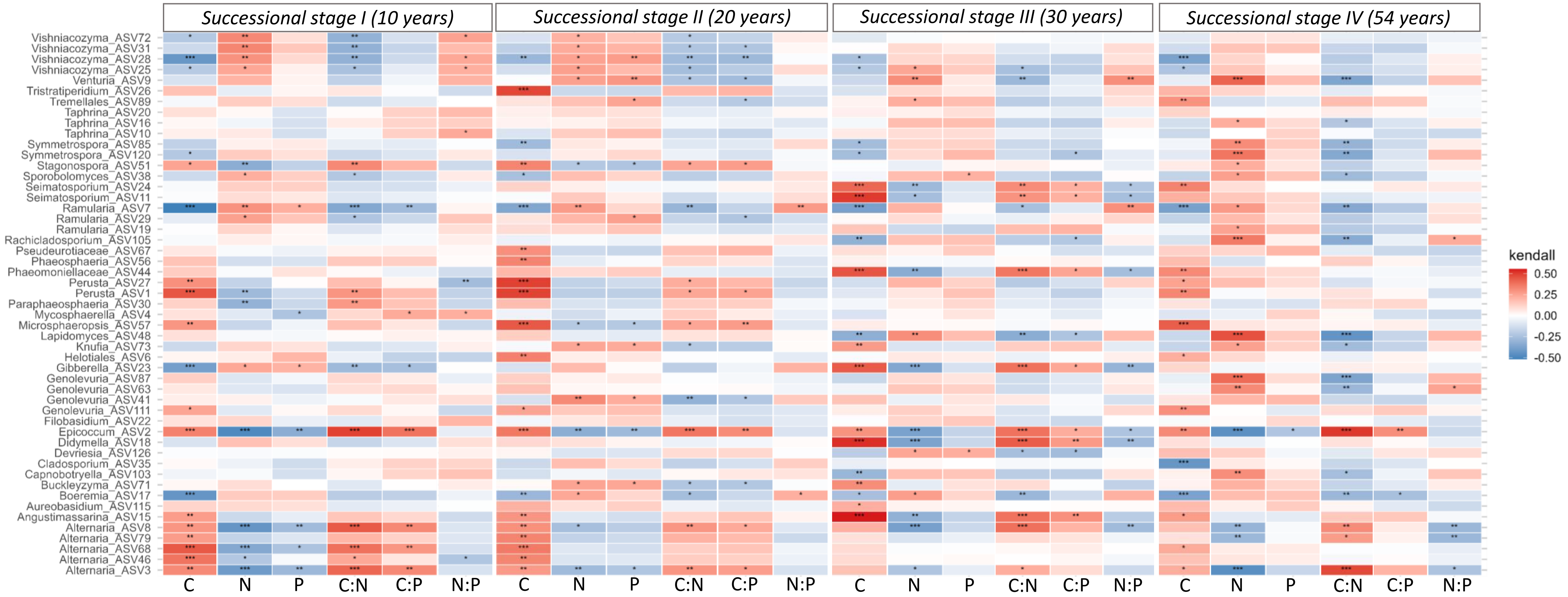
Successional stage I (10 years)
Successional stage II (20 years)
Successional stage III (30 years)
Successional stage IV (54 years)
Vishniacozyma_ASV72
Vishniacozyma_ASV31
Vishniacozyma_ASV28
Vishniacozyma_ASV25
Venturia_ASV9
Tristratiperidium_ASV26
Tremellales_ASV89
Taphrina_ASV20
Taphrina_ASV16
Taphrina_ASV10
Symmetrospora_ASV85
Symmetrospora_ASV120
Stagonospora_ASV51
Sporobolomyces_ASV38
Seimatosporium_ASV24
Seimatosporium_ASV11
Ramularia_ASV7
Ramularia_ASV29
Ramularia_ASV19
Rachicladosporium_ASV105
Pseudeurotiaceae_ASV67
Phaeosphaeria_ASV56
Phaeomoniellaceae_ASV44
Perusta_ASV27
Perusta_ASV1
Paraphaeosphaeria_ASV30
Mycosphaerella_ASV4
Microsphaeropsis_ASV57
Lapidomyces_ASV48
Knufia_ASV73
Helotiales_ASV6
Gibberella_ASV23
Genolevuria_ASV87
Genolevuria_ASV63
Genolevuria_ASV41
Genolevuria_ASV111
Filobasidium_ASV22
Epicoccum_ASV2
Didymella_ASV18
Devriesia_ASV126
Cladosporium_ASV35
Capnobotryella_ASV103
Buckleyzyma_ASV71
Boeremia_ASV17
Aureobasidium_ASV115
Angustimassarina_ASV15
Alternaria_ASV8
Alternaria_ASV79
Alternaria_ASV68
Alternaria_ASV46
Alternaria_ASV3
C
N
P
C:N
C:P
N:P
kendall
0.50
0.25
0.00
-0.25
-0.50

Figure S7

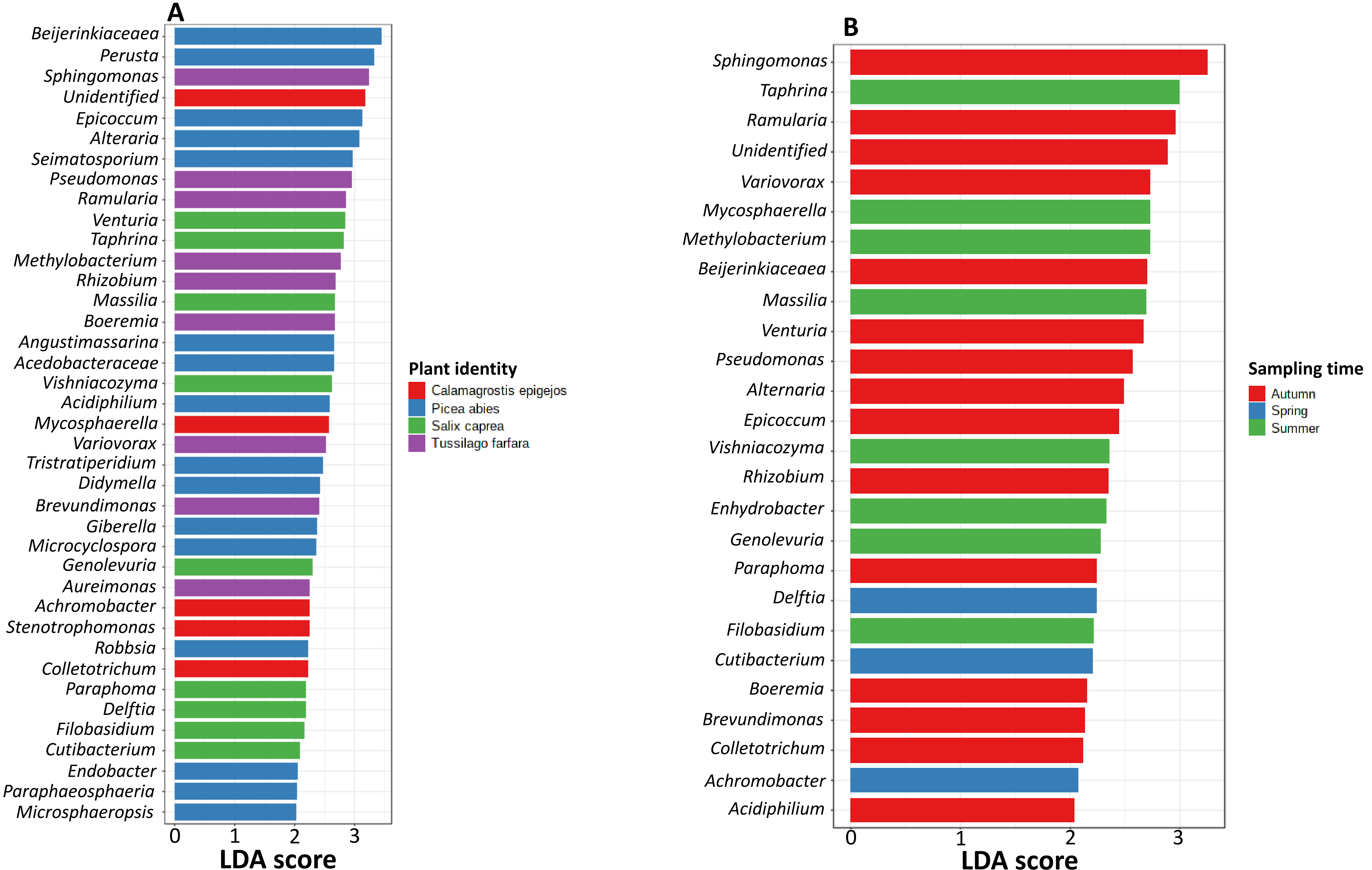
A
Beijerinkiaceaea
Perusta
Sphingomonas
Unidentified
Epicoccum
Alteraria
Seimatosporium
Pseudomonas
Ramularia
Venturia
Taphrina
Methylobacterium
Rhizobium
Massilia
Boeremia
Angustimassarina
Acedobacteraceae
Vishniacozyma
Acidiphilium
Mycosphaerella
Variovorax
Tristratiperidium
Didymella
Brevundimonas
Giberella
Microcyclospora
Genolevuria
Aureimonas
Achromobacter
Stenotrophomonas
Robbsia
Colletotrichum
Paraphoma
Delftia
Filobasidium
Cutibacterium
Endobacter
Paraphaeosphaeria
Microsphaeropsis
0
1
2
3
LDA score
Plant identity
Calamagrostis epigejos
Picea abies
Salix caprea
Tussilago farfara
B
Sphingomonas
Taphrina
Ramularia
Unidentified
Variovorax
Mycosphaerella
Methylobacterium
Beijerinkiaceaea
Massilia
Venturia
Pseudomonas
Alternaria
Epicoccum
Vishniacozyma
Rhizobium
Enhydrobacter
Genolevuria
Paraphoma
Delftia
Filobasidium
Cutibacterium
Boeremia
Brevundimonas
Colletotrichum
Achromobacter
Acidiphilium
0
1
2
3
LDA score
Sampling time
Autumn
Spring
Summer

Figure S8

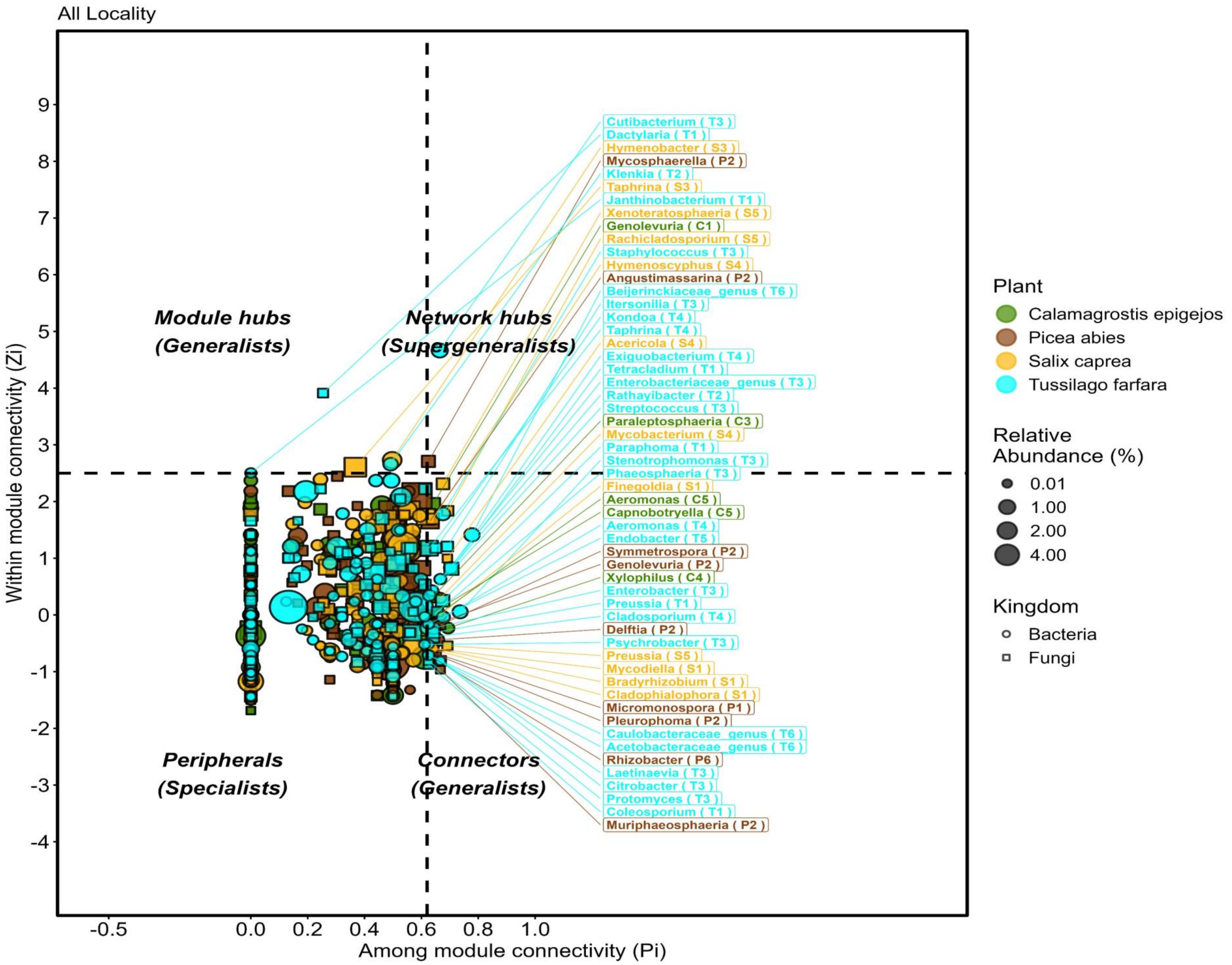
All Locality
Module hubs
(Generalists)
Network hubs
(Supergeneralists)
Peripherals
(Specialists)
Connectors
(Generalists)
Within module connectivity (Zi)
Among module connectivity (Pi)
9
8
7
6
5
4
3
2
1
0
-1
-2
-3
-4
-0.5
0.0
0.2
0.4
0.6
0.8
1.0
Cutibacterium (T3)
Dactylaria (T1)
Hymenobacter (S3)
Mycosphaerella (P2)
Klenkia (T2)
Taphrina (S3)
Janthinobacterium (T1)
Xenoteratosphaeria (S5)
Genolevuria (C1)
Rachicladosporium (S5)
Staphylococcus (T3)
Hymenoscyphus (S4)
Angustimassarina (P2)
Beijerinckiaceae_genus (T6)
Itersonilia (T3)
Kondoa (T4)
Taphrina (T4)
Acericola (S4)
Exiguobacterium (T4)
Tetracladium (T1)
Enterobacteriaceae_genus (T3)
Rathayibacter (T2)
Streptococcus (T3)
Paraleptosphaeria (C3)
Mycobacterium (S4)
Paraphoma (T1)
Stenotrophomonas (T3)
Phaeosphaeria (T3)
Finegoldia (S1)
Aeromonas (C5)
Capnobotryella (C5)
Aeromonas (T4)
Endobacter (T5)
Symmetrospora (P2)
Genolevuria (P2)
Xylophilus (C4)
Enterobacter (T3)
Preussia (T1)
Cladosporium (T4)
Delftia (P2)
Psychrobacter (T3)
Preussia (S5)
Mycodiella (S1)
Bradyrhizobium (S1)
Cladophialophora (S1)
Micromonospora (P1)
Pleurophoma (P2)
Caulobacteraceae_genus (T6)
Acetobacteraceae_genus (T6)
Rhizobacter (P6)
Laetinaevia (T3)
Citrobacter (T3)
Protomyces (T3)
Coleosporium (T1)
Muriphaeosphaeria (P2)
Plant
Calamagrostis epigejos
Picea abies
Salix caprea
Tussilago farfara
Relative
Abundance (%)
0.01
1.00
2.00
4.00
Kingdom
Bacteria
Fungi
