# Supplementary material for: Ecological drift and host filtering jointly structure foliar endophytes during ecosystem development
Source: Environ Microbiome. 2026 May 8;21:83. doi: 10.1186/s40793-026-00906-7 (PMC13321508; doi:10.1186/s40793-026-00906-7)
Supplement: Supplementary file 3 — Supplementary Material 3 [file 40793_2026_906_MOESM3_ESM.docx]

**Table S2.** Physicochemical soil properties across four successional stages (I, II, III, and IV). Values (average ± standard deviation) represent measurements of pH, electrical conductivity (μS), available phosphorus (P – mg kg^-1^), total phosphorus (Total P - mg kg^-1^), total nitrogen (Total N - %), total carbon (Total C - %), carbon-to-nitrogen ratio (C:N), and carbon and nitrogen from microbial biomass (C_mic_, N_mic_ - µg g^-1^).

| **Successional stages** | **pH** | **Conductivity** | **P available** | **Total P** | **Total N** | **Total C** | **C:N ratio** | **C_mic_** | **N_mic_** |
| --- | --- | --- | --- | --- | --- | --- | --- | --- | --- |
| **Location I – 10 yr.** | 8.1 ± 0.1 | 140.5 ± 10.7 | 146.6 ± 76.0 | 1118.5 ± 194.3 | 0.3 ± 0.0 | 6.1 ± 0.6 | 20.8 ± 1.8 | 515.5 ± 198.1 | 81.8 ± 23.7 |
| **Location II – 20 yr.** | 8.0 ± 0.4 | 164.3 ± 24.2 | 74.1 ± 37.0 | 1015.5 ± 230.6 | 0.5 ± 0.2 | 7.7 ± 4.2 | 14.0 ± 3.0 | 357.4 ± 28.9 | 59.1 ± 3.5 |
| **Location III – 30 yr.** | 7.4 ± 0.2 | 199.8 ± 33.9 | 105.8 ± 18.0 | 1250.0 ± 158.4 | 1.1 ± 0.3 | 19.7 ± 5.1 | 17.6 ± 0.3 | 2712.8 ± 2476.8 | 468.1 ± 430.2 |
| **Location IV – 54 yr.** | 7.8 ± 0.1 | 121.5 ± 13.2 | 69.8 ± 10.9 | 1128.3 ± 51.4 | 0.6 ± 0.0 | 8.4 ± 0.8 | 14.6 ± 0.8 | 1227.2 ± 352.1 | 198.4 ± 23.0 |
