# Supplementary material for: Ecological drift and host filtering jointly structure foliar endophytes during ecosystem development
Source: Environ Microbiome. 2026 May 8;21:83. doi: 10.1186/s40793-026-00906-7 (PMC13321508; doi:10.1186/s40793-026-00906-7)
Supplement: Supplementary file 4 — Supplementary Material 4 [file 40793_2026_906_MOESM4_ESM.docx]

**TABLE S3.** Variation partitioning results showing the contribution of explanatory fractions to the variation in bacterial and fungal community structure, predicted functional groups and leaf tissue stoichiometry. Adjusted R² values represent the proportion of variation uniquely explained by each fraction after accounting for shared effects and model complexity. “% of Explained Variation” refers to the proportion of total explained variation attributed to each fraction, while “% of Total Variation” represents the fraction’s contribution to the total variation in the dataset.

|  | **Fraction** | **Adjusted R²** | **% of Explained Variation** | **% of Total Variation** |
| --- | --- | --- | --- | --- |
| **BACTERIA** | Successional stage | 0.179 | 25.1 | 2.5 |
|  | Host identity | 0.361 | 50.6 | 5.1 |
|  | Season | 0.167 | 23.4 | 2.4 |
|  | **Total** | **0.714** | **99.1** | **10.1** |
|  |  |  |  |  |
|  | Essential macronutrients | 0.122 | 19.0 | 1.7 |
|  | Trace metals | 0.086 | 13.4 | 1.2 |
|  | Primary nutrients | 0.128 | 20 | 1.8 |
|  | **Total** | **0.336** | **52.4** | **4,7** |
|  |  |  |  |  |
| **FUNGI** | Successional stage | 0.334 | 39.0 | 4.6 |
|  | Host identity | 0.419 | 49.0 | 5.8 |
|  | Season | 0.092 | 10.7 | 1.3 |
|  | **Total** | **0.845** | **98.7** | **11.7** |
|  |  |  |  |  |
|  | Essential macronutrients | 0.160 | 23.7 | 2.2 |
|  | Trace metals | 0.084 | 12.6 | 1.2 |
|  | Primary nutrients | 0.163 | 24.1 | 2.2 |
|  | **Total** | **0.407** | **60.4** | **5.6** |
|  |  |  |  |  |
| **FUNCTIONAL**  **GENES** | Successional stage | 0.006 | 2.5 | 0.7 |
|  | Host identity | 0.230 | 84.4 | 23.1 |
|  | Season | 0.040 | 14.7 | 4.0 |
|  | **Total** | **0.276** | **100.0** | **27.8** |
|  |  |  |  |  |
| **LEAF**  **STOICHIOMETRY** | Successional stage | 0.024 | 6.0 | 2.5 |
|  | Host identity | 0.306 | 74,.8 | 30.6 |
|  | Season | 0.085 | 21.0 | 8.6 |
|  | **Total** | **0.415** | **100.0** | **41.7** |
