# Supplementary material for: Ecological drift and host filtering jointly structure foliar endophytes during ecosystem development
Source: Environ Microbiome. 2026 May 8;21:83. doi: 10.1186/s40793-026-00906-7 (PMC13321508; doi:10.1186/s40793-026-00906-7)
Supplement: Supplementary file 5 — Supplementary Material 5 [file 40793_2026_906_MOESM5_ESM.docx]

**Table S1.** Molar ratios of carbon:nitrogen (C:N), carbon:phosphorus (C:P), and nitrogen:phosphorus (N:P) in the leaf tissue of four experimental plant species (Calamagrostis epigejos, Picea abies, Salix caprea, and Tussilago farfara) along the gradient of primary succession (Location I, II, III, and IV) sampled at three different time points during the growing season(Spring, Summer, Autumn). Means ± SD are shown (n=6).

|  |  | **C:N** |  |  | **C:P** |  |  | **N:P** |  |
| --- | --- | --- | --- | --- | --- | --- | --- | --- | --- |
|  | Spring | Summer | Autumn | Spring | Summer | Autumn | Spring | Summer | Autumn |
| **Location I** |  |  |  |  |  |  |  |  |  |
| *C. epigejos* | 21.3 ± 4.6 ^A-a^ | 41.7 ± 4.3 ^B-a^ | 30.7 ± 5.6 ^A -a^ | 238.9 ± 72.7 ^A-a^ | 351.1 ± 97.2 ^A-a^ | 276.5 ± 59.0 ^A-a^ | 11.2 ± 2.8 ^A-a^ | 8.4 ± 2.2 ^A-a^ | 9.11 ± 2.06 ^A-a^ |
| *P. abies* | 55.2 ± 6.0 ^A-a^ | 70.3 ± 6.5 ^B-a^ | 58.2 ± 8.2 ^A-a^ | 348.8 ± 87.8 ^A-a,b^ | 394.4 ± 125.0 ^A-a,c^ | 302.2 ± 95.3 ^A-a^ | 6.3 ± 1.4 ^A-a^ | 5.5 ± 1.4 ^A-a^ | 5.0 ± 1.0 ^A-a^ |
| *S. caprea* | 10.2 ± 0.5 ^A-a^ | 25.1 ± 3.5 ^B-a^ | 34.1 ± 3.7 ^C-a^ | 63.3 ± 7.6 ^A-a^ | 274.1 ± 38.1 ^B-a^ | 322.6 ± 49.8 ^B-a^ | 6.1 ± 0.4 ^A-a^ | 11.0 ± 1.6 ^B-a^ | 9.4 ± 1.0 ^B-a^ |
| *T. farfara* | 26.0 ± 7.2 ^A-c^ | 24.3 ± 2.2 ^A-a^ | 26.7 ± 6.8 ^A-a^ | 129.5 ± 29.3 ^A-a^ | 199.5 ± 64.4 ^B-a^ | 193.2 ± 40.5 ^B-a^ | 5.0 ± 0.9 ^A-a^ | 8.3 ± 3.2 ^B-a,b^ | 7.5 ± 2.0 ^B-a^ |
|  |  |  |  |  |  |  |  |  |  |
| **Location II** |  |  |  |  |  |  |  |  |  |
| *C. epigejos* | 28.1 ± 5.0 ^A-a^ | 55.4 ± 6.7 ^B-b^ | 47.8 ± 11.8 ^B-b^ | 251.6 ± 21.7 ^A-a^ | 343.6 ± 130.8 ^A-a^ | 333.7 ± 113.7 ^A-a^ | 9.1 ± 2.0 ^A-a,b^ | 6.1 ± 2.1 ^B-a,b^ | 6.9 ± 1.8 ^A,B-a,b^ |
| *P. abies* | 61.0 ± 8.8 ^A,B-a^ | 69.7 ± 7.1 ^B-a^ | 56.3 ± 13.9 ^A-a^ | 430.1 ± 97.5 ^A-a^ | 375.5 ± 53.3 ^A,B-a^ | 328.6 ± 65.8 ^B-a^ | 6.9 ± 0.8 ^A-a^ | 5.4 ± 0.9 ^B-a^ | 5.9 ± 1.0 ^B-a^ |
| *S. caprea* | 10.3 ± 1.4 ^A-a^ | 29.6 ± 3.0 ^B-a^ | 36.2 ± 10.0 ^C-a^ | 62.9 ± 8.5 ^A-a^ | 187.0 ± 18.1 ^B-b^ | 192.0 ± 65.5 ^B-b^ | 6.1 ± 0.5 ^A,B-a^ | 6.3 ± 0.3 ^A-b^ | 5.2 ± 0.7 ^B-b^ |
| *T. farfara* | 17.2 ± 2.6 ^A-a,b^ | 23.4 ± 1.8 ^A,B-a^ | 29.6 ± 5.8 ^B-a,b^ | 185.4 ± 40.3 ^A-a,b^ | 206.8 ± 51.5 ^A-a^ | 252.7 ± 101.7 ^A-a^ | 11.3 ± 4.7 ^A-b^ | 8.7 ± 1.6 ^A-a^ | 8.3 ± 1.9 ^A-a^ |
|  |  |  |  |  |  |  |  |  |  |
| **Location III** |  |  |  |  |  |  |  |  |  |
| *C. epigejos* | 27.8 ± 2.7 ^A-a^ | 35.1 ± 5.0 ^B-a^ | 37.6 ± 5.4 ^B-a,b^ | 193.3 ± 79.1 ^A-a^ | 205.5 ± 30.4 ^A-b^ | 253.4 ± 35.6 ^A-a^ | 7.0 ± 3.0 ^A-b,c^ | 5.8 ± 0.8 ^A-b,c^ | 6.8 ± 1.4 ^A-b^ |
| *P. abies* | 54.2 ± 3.3 ^B-a^ | 49.0 ± 2.5 ^A,B-b^ | 41.7 ± 1.5 ^A-b^ | 328.4 ± 26.5 ^A-b^ | 250.1 ± 20.2 ^B-b^ | 220.3 ± 13.2 ^B-b^ | 6.2 ± 0.5 ^A-a^ | 5.0 ± 0.3 ^B-a^ | 5.2 ± 0.4 ^B-a^ |
| *S. caprea* | 13.4 ± 0.9 ^A-b^ | 23.4 ± 1.5 ^B-a^ | 31.9 ± 3.7 ^C-a^ | 92.9 ± 4.7 ^A-b^ | 214.6 ± 40.8 ^B-b^ | 274.3 ± 65.6 ^B-a,b^ | 6.6 ± 0.3 ^A-a^ | 9.1 ± 1.5 ^B-a,c^ | 8.6 ± 2.0 ^B-a^ |
| *T. farfara* | 15.4 ± 3.1 ^A-a^ | 23.8 ± 2.4 ^B-a^ | 29.1 ± 4.2 ^B-a,b^ | 154.1 ± 68.7 ^A-a,b^ | 185.0 ± 41.8 ^A-a^ | 209.8 ± 35.3 ^A-a^ | 9.6 ± 3.1 ^A-b^ | 7.8 ± 2.0 ^A-a,b^ | 7.2 ± 0.5 ^A-a^ |
|  |  |  |  |  |  |  |  |  |  |
| **Location IV** |  |  |  |  |  |  |  |  |  |
| *C. epigejos* | 22.8 ± 5.1 ^A-a^ | 38.3 ± 4.7 ^B-a^ | 29.6 ± 4.7 ^A,B-a^ | 129.1 ± 53.1 ^A-b^ | 252.4 ± 68.1 ^B-a,b^ | 264.0 ± 13.4 ^B-a^ | 5.9 ± 1.1 ^A-c^ | 6.6 ± 1.9 ^A-a,c^ | 9.0 ± 1.3 ^B-a,b^ |
| *P. abies* | 59.1 ± 8.1 ^B-a^ | 52.2 ± 5.7 ^A,B-b^ | 43.6 ± 5.3 ^A-b^ | 409.4 ± 66.0 ^B-a^ | 288.1 ± 71.1 ^B-b,c^ | 263.7 ± 31.8 ^B-a,b^ | 6.9 ± 1.3 ^A-a^ | 5.4 ± 1.0 ^B-a^ | 6.1 ± 1.0 ^A,B-a^ |
| *S. caprea* | 13.6 ± 1.9 ^A-b^ | 27.9 ± 2.8 ^B-a^ | 34.4 ± 5.2 ^B-a^ | 144.5 ± 139.7 ^A-b^ | 211.7 ± 36.5 ^A,B-b^ | 256.9 ± 66.8 ^B-a,b^ | 6.3 ± 0.6 ^A-a^ | 7.6 ± 1.5 ^A-b,c^ | 7.6 ± 2.6 ^A-a,b^ |
| *T. farfara* | 24.2 ± 3.6 ^A-b,c^ | 29.9 ± 5.7 ^A,B-a^ | 35.9 ± 6.0 ^B-b^ | 276.4 ± 120.6 ^A-b^ | 161.6 ± 77.0 ^A-a^ | 221.7 ± 74.2 ^A-a^ | 12.6 ± 3.9 ^A-b^ | 5.3 ± 2.3 ^B-b^ | 6.1 ± 1.3 ^B-a^ |

Different capital letters within the same row designate statistically significant seasonal difference (p < 0.05) for a given plant species. Different lower case letters within the same column designate site-specific statistically significant differences (p < 0.05) for a given plant species.
