# Supplementary material for: Ecological drift and host filtering jointly structure foliar endophytes during ecosystem development
Source: Environ Microbiome. 2026 May 8;21:83. doi: 10.1186/s40793-026-00906-7 (PMC13321508; doi:10.1186/s40793-026-00906-7)
Supplement: Supplementary file 7 — Supplementary Material 7 [file 40793_2026_906_MOESM7_ESM.docx]

**Table S6.** Bacterial richness (Chao1 ± SD (CV)) for four plant species (Calamagrostis epigejos - CE, Picea abies - PA, Salix caprea – SC, and Tussilago farfara - TF) in four different locations (I, II, III and IV). Different lowercase letter in the same row and different uppercase letter in the same column means significant difference (p < 0.05).

| **Plants** | **I** | **II** | **III** | **IV** |
| --- | --- | --- | --- | --- |
| **CE** | 21.77 ± 14.61 (67.10) **^a - A^** | 24.41 ± 18.00 (73.76) **^a - A^** | 65.75 ± 63.45 (96.50) **^b - A^** | 27.80 ± 15.56 (55.99) **^a - A^** |
| **PA** | 28.50 ± 12.75 (44.76) **^a - A,B^** | 29.00 ± 10.52 (36.30) **^b - A^** | 105.50 ± 57.88 (54.87) **^b - B^** | 70.11 ± 29.83 (42.54) **^b - B^** |
| **SC** | 43.61 ± 24.46 (56.09) **^a - B^** | 36.70 ± 27.65 (75.33) **^a - A^** | 114.39 ± 86.01 (75.19) **^b - B^** | 70.68 ± 58.02 (82.09) **^a – B,C^** |
| **TF** | 81.52 ± 52.61 (64.53) **^a - C^** | 82.93 ± 51.26 (61.81) **^a - B^** | 76.05 ± 39.53 (51.98) **^a - A^** | 95.88 ± 56.83 (59.27) **^a – C^** |

Different capital letters within the same row designate statistically significant difference (p < 0.05) for a given plant species. Different lower-case letters within the same column designate site-specific statistically significant differences (p < 0.05).

**Table S7.** Bacterial diversity (Shannon ± SD (CV)) for four plant species (Calamagrostis epigejos - CE, Picea abies - PA, Salix caprea – SC, and Tussilago farfara - TF) in four different locations (I, II, III and IV). Different lowercase letter in the same row and different uppercase letter in the same column means significant difference (p < 0.05).

| **Plants** | **I** | **II** | **III** | **IV** |
| --- | --- | --- | --- | --- |
| **CE** | 2.20 ± 0.82 (37.19) **^a – A^** | 2.37 ± 0.72 (30.67) **^a - A^** | 2.73 ± 1.15 (42.19) **^b - A^** | 2.41 ± 0.83 (34.79) **^a - A^** |
| **PA** | 2.28 ± 0.63 (17.26) **^a - A^** | 2.26 ± 0.51 (28.83) **^a – A^** | 3.57 ± 0.71 (19.96) **^b - B^** | 3.24 ± 0.36 (11.29) **^b - B^** |
| **SC** | 2.63 ± 0.84 (32.18) **^a – A,B^** | 2.74 ± 0.38 (14.14) **^a – A,C^** | 3.49 ± 0.72 (20.86) **^b - B^** | 3.05 ± 0.64 (21.11) **^b - B^** |
| **TF** | 2.83 ± 0.74 (26.12) **^a – B^** | 2.93 ± 0.87 (29.70) **^a – B^** | 2.95 ± 0.58 (19.68) **^a - A^** | 3.17 ± 0.65 (20.76) **^a - B^** |

Different capital letters within the same row designate statistically significant difference (p < 0.05) for a given plant species. Different lower-case letters within the same column designate site-specific statistically significant differences (p < 0.05).

**Table S8.** Fungal richness (Chao1 ± SD (CV)) for four plant species (Calamagrostis epigejos - CE, Picea abies - PA, Salix caprea – SC, and Tussilago farfara - TF) in four different locations (I, II, III and IV). Different lowercase letter in the same row and different uppercase letter in the same column means significant difference (p < 0.05).

| **Plants** | **I** | **II** | **III** | **IV** |
| --- | --- | --- | --- | --- |
| **CE** | 16.27 ± 10.86 (66.72) **^a - A^** | 31.29 ± 28.59 (91.37) **^a - A^** | 56.81 ± 54.96 (96.74) **^b - A^** | 32.80 ± 29.43 (89.72) **^a,b - A^** |
| **PA** | 93.62 ± 25.87 (27.63) **^a,b - B^** | 71.27 ± 20.40 (28.62) **^a - B^** | 99.50 ± 33.13 (33.30) **^b - B^** | 118.72 ± 30.72 (25.88) **^b - B^** |
| **SC** | 59.72 ± 49.17 (82.34) **^a - C^** | 51.76 ± 47.36 (91.49) **^a - A,B^** | 64.23 ± 38.33 (59.67) **^a - A^** | 46.66 ± 40.68 (87.18) **^a - A^** |
| **TF** | 56.33 ± 59.11 (104.94) **^a - C^** | 72.75 ± 56.80 (78.07) **^a - B^** | 52.88 ± 50.51 (95.51) **^a - A^** | 81.70 ± 55.55 (67.99) **^b - B^** |

Different capital letters within the same row designate statistically significant difference (p < 0.05) for a given plant species. Different lower-case letters within the same column designate site-specific statistically significant differences (p < 0.05).

**Table S9.** Fungal diversity (Shannon ± SD (CV)) for four plant species (Calamagrostis epigejos - CE, Picea abies - PA, Salix caprea – SC, and Tussilago farfara - TF) in four different locations (I, II, III and IV). Different lowercase letter in the same row and different uppercase letter in the same column means significant difference (p < 0.05).

| **Plants** | **I** | **II** | **III** | **IV** |
| --- | --- | --- | --- | --- |
| **CE** | 1.60 ± 0.73 (45.98) **^a - A^** | 1.69 ± 0.94 (55.97) **^a - A^** | 2.41 ± 0.94 (39.23) **^b - A^** | 1.59 ± 0.73 (46.19) **^a - A^** |
| **PA** | 2.82 ± 0.36 (13.00) **^a - B^** | 2.29 ± 0.40 (17.41) **^b - B^** | 2.96 ± 0.49 (16.63) **^a,c - B^** | 3.15 ± 0.51 (16.42) **^a - B^** |
| **SC** | 2.72 ± 0.78 (28.84) **^a - B^** | 2.35 ± 1.11 (47.54) **^a - B^** | 2.65 ± 0.64 (24.41) **^a - A,B^** | 2.65 ± 1.03 (39.07) **^a - B^** |
| **TF** | 2.26 ± 1.02 (45.38) **^a - C^** | 2.98 ± 0.94 (31.64) **^b - C^** | 2.54 ± 0.73 (28.97) **^a - A,B^** | 3.13 ± 0.83 (26.49) **^b - B^** |

Different capital letters within the same row designate statistically significant difference (p < 0.05) for a given plant species. Different lower-case letters within the same column designate site-specific statistically significant differences (p < 0.05).
