# Supplementary material for: Ecological drift and host filtering jointly structure foliar endophytes during ecosystem development
Source: Environ Microbiome. 2026 May 8;21:83. doi: 10.1186/s40793-026-00906-7 (PMC13321508; doi:10.1186/s40793-026-00906-7)
Supplement: Supplementary file 8 — Supplementary Material 8 [file 40793_2026_906_MOESM8_ESM.docx]

**Table S10.** Bacterial and fungal turnover (mean ± SD) for four plant species (Calamagrostis epigejos, Picea abies, Salix caprea and Tussilago farfara) in two different time point: from spring to summer (T1) and from summer to autumn (T2) across the four different locations (I, II, III and IV). Different lowercase letter in the same row and different uppercase letter in the same column means significant difference (p < 0.05).

|  |  | **BACTERIAL TURNOVER** | | **FUNGAL TURNOVER** | |
| --- | --- | --- | --- | --- | --- |
| **Location** | **Plants** | **T_1_** | **T_2_** | **T_1_** | **T_2_** |
| **I** | *Calamagrostis epigejos* | 0.748 ± 0.119 **^a – A – 1^** | 0.700 ±0.78 **^a - A – 1^** | 0.753 ± 0.135 **^a - A – 1^** | 0.701 ± 0.084 **^a – A – 1^** |
| **I** | *Picea abies* | 0.687 ± 0.046 **^a – A – 1^** | 0.629 ± 0.029 **^b – A,B – 1^** | 0.455 ± 0.063 **^a - B – 1^** | 0.431 ± 0.047 **^a – B – 1,2,3^** |
| **I** | *Salix caprea* | 0.704 ± 0.079 **^a – A – 1^** | 0.606 ± 0.085 **^a – A – 1^** | 0.800 ± 0.104 **^a - A – 1^** | 0.490 ± 0.062 **^b – B,C – 1^** |
| **I** | *Tussilago farfara* | 0.784 ± 0.103 **^a – A – 1^** | 0.446 ± 0.035 **^b – B – 1^** | 0.833 ± 0.068 **^a - A – 1,2,3^** | 0.653 ± 0.131 **^b – A,C – 1^** |
| **II** | *Calamagrostis epigejos* | 0.705 ± 0.097 **^a – A,B,C – 1^** | 0.670 ±0.138 **^a - A – 1,2^** | 0.816 ± 0.078 **^a - A – 1^** | 0.610 ± 0.085 **^b - A – 1,2^** |
| **II** | *Picea abies* | 0.615 ± 0.077 **^a – B – 1,2^** | 0.595 ± 0.091 **^a – A,B – 1^** | 0.466 ± 0.044 **^a - B – 1^** | 0.471 ± 0.075 **^a – B,C – 2^** |
| **II** | *Salix caprea* | 0.742 ± 0.036 **^a – C – 1^** | 0.618 ± 0.120 **^b – A,B – 1^** | 0.822 ± 0.096 **^a - A – 1^** | 0.560 ± 0.112 **^b – A,C – 1^** |
| **II** | *Tussilago farfara* | 0.716 ± 0.081 **^a – A,B,C – 1,2^** | 0.473 ± 0.107 **^b – B – 1,2^** | 0.799 ± 0.126 **^a - A – 1,2,3^** | 0.564 ± 0.086 **^b – A,C – 1^** |
| **III** | *Calamagrostis epigejos* | 0.813 ± 0.038 **^a – A – 1^** | 0.554 ±0.138 **^b - A – 2^** | 0.828 ± 0.073 **^a - A – 1^** | 0.624 ± 0.048 **^b - A – 1,2^** |
| **III** | *Picea abies* | 0.547 ± 0.116 **^a – B – 2^** | 0.510 ± 0.091 **^a – A – 1^** | 0.424 ± 0.051 **^a - B – 1^** | 0.380 ± 0.049 **^a – B – 3^** |
| **III** | *Salix caprea* | 0.715 ± 0.120 **^a – A,C – 1^** | 0.539 ± 0.120 **^b – A – 1^** | 0.738 ± 0.165 **^a - A – 1^** | 0.536 ± 0.087 **^b – A – 1^** |
| **III** | *Tussilago farfara* | 0.644 ± 0.056 **^a – B,C – 2^** | 0.525 ± 0.107 **^b – A – 2^** | 0.758 ± 0.049 **^a - A – 2^** | 0.603 ± 0.074 **^b – A – 1^** |
| **IV** | *Calamagrostis epigejos* | 0.797 ± 0.060 **^a – A – 1^** | 0.644 ±0.021 **^b – A – 1,2^** | 0.863 ± 0.035 **^a - A – 1^** | 0.556 ± 0.106 **^b – A,B,C – 2^** |
| **IV** | *Picea abies* | 0.568 ± 0.069 **^a – B – 2^** | 0.583 ± 0.099 **^a – A – 1^** | 0.449 ± 0.036 **^a - B – 1^** | 0.440 ± 0.052 **^a – B – 1,2,3^** |
| **IV** | *Salix caprea* | 0.702 ± 0.072 **^a – A – 1^** | 0.554 ± 0.050 **^b – A,B – 1^** | 0.817 ± 0.052 **^a - A – 1^** | 0.593 ± 0.136 **^b – C – 1^** |
| **IV** | *Tussilago farfara* | 0.697 ± 0.061 **^a – B – 1,2^** | 0.463 ± 0.059 **^b – B – 1,2^** | 0.843 ± 0.098 **^a - A – 3^** | 0.522 ± 0.062 **^b – A,B,C – 1^** |

Different capital letters within the same row designate statistically significant difference (p < 0.05) for a given plant species.

Different lower-case letters within the same location designate statistically significant difference (p < 0.05) for a given plant species between the two time points (T_1_ and T_2_).

Different number within the same time period (column) designate statistically significant difference (p < 0.05) for a given plant species comparing all four locations.
